# ﻿Eighteen new species of Neotropical Costaceae (Zingiberales)

**DOI:** 10.3897/phytokeys.222.87779

**Published:** 2023-03-22

**Authors:** Paul J. M. Maas, Hiltje Maas-van de Kamer, Thiago André, David Skinner, Eugenio Valderrama, Chelsea D. Specht

**Affiliations:** 1 Naturalis Biodiversity Centre, Botany, P.O. Box 9517, 2300 RA Leiden, Netherlands Naturalis Biodiversity Centre Leiden Netherlands; 2 Universidade de Brasília, Departamento de Botânica, Campus Universitário Darcy Ribeiro, Asa Norte, Brasília (DF), Brazil Universidade de Brasília Brasília Brazil; 3 Le Jardin Ombragé, Tallahassee, (Private botanical garden, Botanic Gardens Conservation International – BGCI – registration ID 50148), Florida, USA Le Jardin Ombragé Tallahassee United States of America; 4 Cornell University, Section of Plant Biology and the L.H.Bailey Hortorium, School of Integrative Plant Science, Ithaca, NY, USA Cornell University Ithaca United States of America

**Keywords:** *Chamaecostus*, *Costus*, *Dimerocostus*, Monocots, spiral ginger

## Abstract

In preparation for a full taxonomic revision of the Neotropical genera of Costaceae (i.e., *Chamaecostus*, *Costus*, *Dimerocostus*, and *Monocostus*), we present the description of 17 new species of Neotropical *Costus* and one new species of the Neotropic endemic genus *Chamaecostus* with notes on their distribution and ecology, vernacular names (when known), and diagnostic characters for identification. Distribution maps are included for all species, and each description is accompanied by photographic plates illustrating diagnostic characters.

## ﻿Introduction

The Neotropical members of the family Costaceae (Zingiberales) comprise two separate evolutionary lineages of species included in genera *Costus*, *Chamaecostus*, *Dimerocostus* and *Monocostus*. Originally considered a subfamily of the Zingiberaceae, they were first treated comprehensively in Floral Neogropica monographs No. 8 Costoideae (Zingiberaceae) and Monograph No. 18 Costoideae (Additions) (Maas 1972, 1977) and as part of the Flora of Suriname (Maas 1979). Species pertaining to the genus *Chamaecostus*, included in these monographs as CostussubgenusCadalvena, were subsquently removed from *Costus* based on phylogenetic analyses demonstrating that they are in fact more closely related to *Monocostus* and *Dimerocostus* than to other species of *Costus* (Specht et al. 2001; Specht 2006a; Specht and Stevenson 2006). Of these genera, *Chamaecostus*, *Monocostus* and *Dimerocostus* are endemic to the Neotropics while *Costus* contains species with African distributions that appear to be ancestral to a clade of Neotropical species (Specht and Stevenson 2006; Specht 2006a, b; Salzman et al. 2015).

Since Maas’ monographic work based largely on observations of herbarium collections, novel observations from extensive field work and herbarium research have resulted in the publication of 18 additional species by Paul Maas (Maas 1976) and Hiltje Maas-van de Kamer (Maas and Maas-van de Kamer 1990, 1997): *Costusasplundii*, *C.asteranthus*, *C.beckii*, *C.cordatus*, *C.cupreifolius*, *C.curvibracteatus*, *C.glaucus*, *C.leucanthus*, *C.nitidus*, *C.osae*, *C.plicatus*, *C.plowmanii*, *C.productus* (to be placed in *C.juruanus*), *C.ricus*, *C.vargasii*, *C.varzearum*, *C.vinosus*, and *C.wilsonii*. A few additional species have been published by other taxonomists, including García-Mendoza and Ibarra-Manríquez (1991), *Costusdirzoi*; Salinas et al. (2007), *Costusfissicalyx*; Salinas and Betancur (2004), *Dimerocostuscryptocalyx*; and the most recent by Pessoa et al. (2020), *Costusatlanticus*.

Recent years have also seen a rise in ecological and evolutionary studies on Costaceae, which have greatly impacted our interpretation of phenotypic patterns and evolutionary processes leading to a better understanding of species delimitations and phylogenetic relationships among taxa (Sakai et al. 1999; Specht et al. 2001; Kay and Schemske 2003; Kay et al. 2005; Kay 2006; Specht 2006a, b; Specht and Stevenson 2006; Araújo and Oliveira 2007; Kay and Schemske 2008; Yost and Kay 2009; Bartlett and Specht 2010; Specht et al. 2012; Surget-Groba and Kay 2013; Almeida et al. 2015; André et al. 2015; Morioka et al. 2015; Salzman et al. 2015; André et al. 2016; Bergamo et al. 2016; Valderrama et al. 2020; Vargas et al. 2020; André et al. 2022). These studies have helped to tease apart species complexes, identify cryptic variation, define biogeographic patterns and pollinator relationships, understand aspects of floral developmental evolution, and explore the tempo and mode of speciation and diversification across the neotropical lineages.

In the present publication, 17 new species of *Costus* and one new species of *Chamaecostus* are described. We include novel character observations made in recent years, including differences in the orientation of the flower relative to the inflorescence, a character that is variable between but consistent within species of *Costus* and thus useful for identification. Floral orientation may be a key feature in defining pollinator preferences and efficiencies (Fenster et al. 2009), and future studies will investigate possible functional significance. In a later study, we will incorporate these new species into a complete revision for all Neotropical Costaceae, including keys to all species, updated distribution maps, photographs with diagnostic characters, a complete index of exsiccatae (comprising ca. 11.000 entries) and a phylogenetic hypothesis to demonstrate evolutionary relationships.

## ﻿Materials and methods

Material from ca. 150 herbaria was consulted for this study. A complete list of specimens examined will be provided in the forthcoming revision (Part II).

In addition, extensive field observations and collections have contributed to this study, supporting our understanding of species distributions and providing opportunities to photograph and observe key traits for distinguishing among taxa, including the colour of floral organs, orientation of the flower relative to the inflorescence (abaxial, erect, adaxial), colour of bracts, and colouration patterns of vegetative material. Field work was led by Paul and Hiltje Maas, with all co-authors participating. Dave Skinner visited most Neotropical countries and made essential observations on this group, particularly visiting type localities and taking detailed photographs, contributing to our understanding of the distribution of characters among taxa. Skinner’s observations have been recorded in iNaturalist and are available at www.inaturalist.org/observations/selvadero. Living material is available in cultivation at the Fairchild Tropical Gardens in Miami, Florida, USA.

## ﻿Taxonomy

### ﻿ Chamaecostus manausensis

Taxon classificationPlantaeZingiberalesCostaceae

Maas & H.Maas sp. nov.

7F707B90-48AD-57E6-8065-3950D4D9487C

urn:lsid:ipni.org:names:77316081-1

#### Diagnosis.

**Figure 1.**
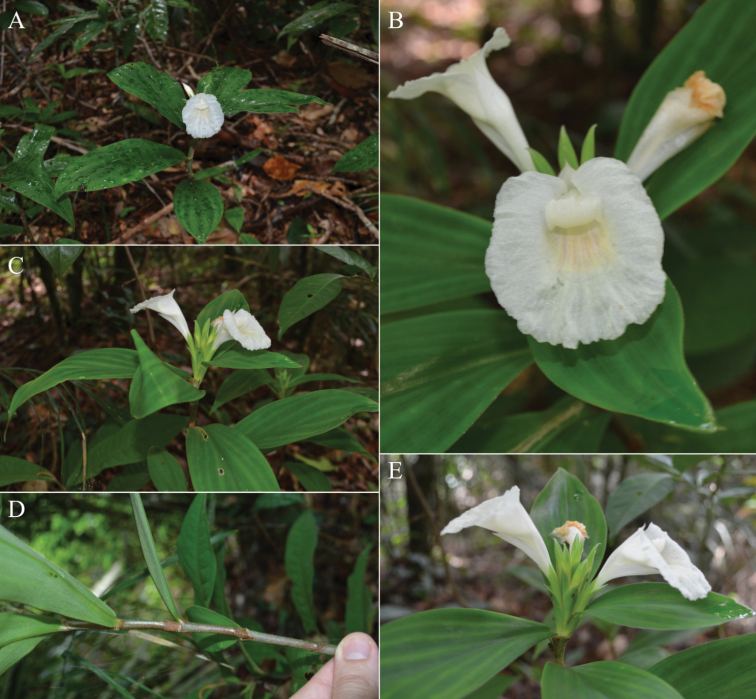
*Chamaecostusmanausensis* Maas & H.Maas **A** plant in habitat Floresta Nacional do Tapajós, Brazil **B** flower showing the non-fimbriate margin of the labellum **C, E** inflorescence narrowly cylindric instead of broadly obovoid **D** sheath and ligules. Photos **A–E** by Thiago André.

*Chamaecostusmanausensis* sp. nov. (Fig. 1) looks quite similar to *C.congestiflorus* and has been regularly confused with that species but can be distinguished by a narrowly cylindric (instead of broadly obovoid) inflorescence and a non-fimbriate labellum.

#### Type.

Brazil. Amazonas: Mauá Road, 22 Mar 1971, *Prance*, *Coêlho*, *Kubitzki & Maas 11529* (holotype: U 2 sheets: U1219716 & U1219717; isotypes: BR, COL128233, CR, DAV, E, F175333, F1842269, GH, INPA, K, MO, NY, P, S, US, VEN, W).

#### Description.

***Herb*** 0.4–1 m tall. ***Leaves*** sheaths 2–15 mm diam; ligule 1–2 mm long; petiole 0–5 mm long; sheaths, ligule and petiole densely puberulous; lamina 13–25 × 3–6 cm, narrowly elliptic, adaxially sparsely puberulous to glabrous, abaxially sometimes reddish-purple, sparsely to densely puberulous, particularly the primary vein, base obtuse, apex acuminate, acumen 5–15 mm long. ***Inflorescence*** 4–10 × 1.5–3 cm, up to ca. 13 × 4 cm in fruit, narrowly cylindric, terminating a leafy shoot; bracts, bracteoles, calyx, ovary, and capsule rather densely to densely puberulous; bracts 2.5–3.5 × 0.3–0.5 cm, narrowly triangular, green, chartaceous, apex often mucronate, callus 3–6 mm long; bracteole 15–20 mm long, tubular, often deeply split on the abaxial side, 2-lobed, lobes 3–7 mm long, deltate. ***Flowers*** calyx 20–33 mm long, green, tube often deeply split during anthesis, lobes 5–10 mm long, subulate; corolla 50–65 mm long, cream, densely puberulous, lobes 30–40 mm long narrowly, obovate; labellum ca. 35 × 40 mm, cream, distal edge horizontally spreading, broadly angular-obovate, margin crenulate; stamen 25–30 × 10 mm, cream, apex reflexed, irregularly dentate, anther 5–6 mm long. ***Capsules*** 11–13 mm long, ellipsoid.

#### Distribution.

Brazil (Amazonas, Pará) (Fig. 21A).

#### Habitat and ecology.

In terra-firme rainforests or campinarana vegetation, on sandy or clayey soils, at elevations of about sea level. Flowering and fruiting: October to March, July.

#### Etymology.

This species is named “manausensis” after the city of Manaus, State of Amazonas, Brazil, where this species is most commonly distributed.

#### Paratypes.

**Brazil. Amazonas**: Reserva Florestal Ducke, km 26 of Manaus-Itacoatiara road, 1 Dec 1994, *Assunção 107* (INPA, U), 5 Jan 1995, *Costa et al. 73* (INPA, U), 12 Dec 1996, *568* (INPA, U), 10 Oct. 1995, *Miralha & Maas 296* (INPA, U), 26 Jan 1995, *Nascimento & Silva 726* (INPA, U); Cachoeira Alta do Tarumã, Manaus, 7 Feb 1930, *Ducke RB 25622* (U); Rio Maués-Acú, across from Maués, Mun. Maués, 21 Jul 1983, *Hill et al. 13138* (INPA, MG, NY, RB, US). **Pará** Floresta Nacional do Tapajós, comunidade Jamaraquá, *Mortati 92* (HSTM).

#### Notes.

For additional field data, see Costa et al. (2011), where this species has been treated under the name *Costuscongestiflorus*.

### ﻿ Costus alfredoi

Taxon classificationPlantaeZingiberalesCostaceae

Maas & H.Maas sp. nov.

9C3F9D55-1EA1-561D-9D8D-56211BEDCC2A

urn:lsid:ipni.org:names:77316083-1

#### Diagnosis.

**Figure 2.**
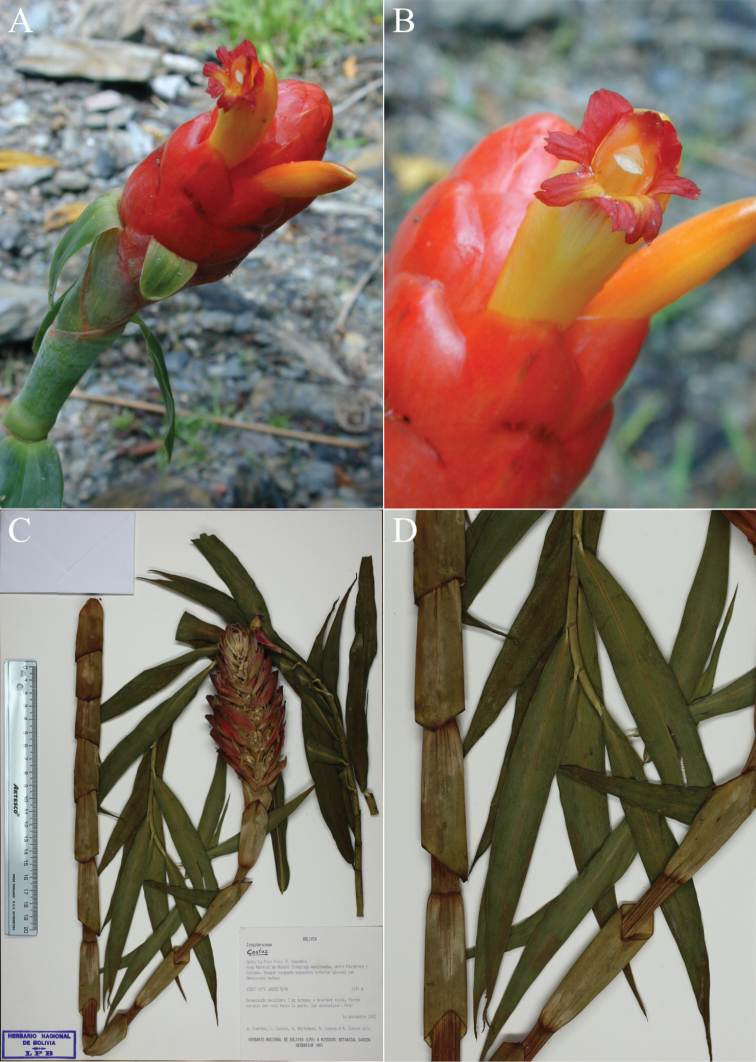
*Costusalfredoi* Maas & H.Maas **A** inflorescence terminal on a leafless shoot **B** flower **C** holotype from Bolivia, La Paz **D** detail from holotype showing narrow, grass-like leaves and short ligule. (**A–D** from *Fuentes et. al. 6216*). Photos by Alfredo Fuentes Claros.

*Costusalfredoi* sp. nov. (Fig. 2) is recognizable by an extremely small ligule (<1 mm long), a “basal” inflorescence (inflorescence terminates a short, leafless shoot and thus is positioned at the base of the plant) with red, unappendaged bracts and yellow, tubular flowers, an almost complete lack of indument, and very narrow, grass-like leaves. It shares the last two features with *C.vargasii* but differs by a shorter ligule (<1 mm vs. 5–35 mm long) and a much longer calyx (15–16 vs. 6–10 mm) and bracteole (25–27 vs. 12–22 mm).

#### Type.

Bolivia, La Paz: Prov. Bautista Saavedra, Area Natural de Manejo Integrado Apolobamba, between Paujeyuyo and Calzada, 1140 m, 16 Nov 2003, *Fuentes Claros*, *Cayola*, *Whitehead & Cuevas 6216* (holotype: MO2098346; isotypes: U1212213 & U1212214).

#### Description.

***Herb*** ca. 2 m tall. ***Leaves*** sheaths 4–5 mm diam; ligule obliquely truncate, <1 mm long; petiole 5–7 mm long; sheaths, ligule and petiole glabrous; lamina linear, 15–23 × 1.5–2.5 cm, adaxially and abaxially glabrous, except for some hairs along the margin, base acute to obtuse, apex long-acute. ***Inflorescence*** ovoid, 11–12 × 5–6 cm, terminating a leafless shoot 50–85 cm long, sheaths obliquely truncate, 6–8 cm long, glabrous; bracts, bracteole, calyx, ovary, and capsule glabrous; bracts red, coriaceous, ovate, 3–4 × 1.5–2.5 cm, apex obtuse, callus 5–7 mm long; bracteole boat-shaped, 25–27 mm long. ***Flowers*** abaxially oriented; calyx 15–16 mm long, lobes shallowly ovate-triangular, 2–3 mm long; corolla yellow to orange, ca. 50 mm long, glabrous, lobes narrowly elliptic, ca. 30 mm long; labellum yellow, lateral lobes rolled inwards and forming a curved tube ca. 10 mm diam, oblong-elliptic when spread out, ca. 35 × 15 mm, middle lobe with yellow honey mark, 5–7–lobulate, lobules red, 5–6 mm long; stamen yellow, ca. 35 × 7 mm, slightly exceeding the labellum, apex red, slightly dentate, anther length not measured. ***Capsule*** ellipsoid, ca. 15 mm long.

#### Distribution.

Bolivia (La Paz) (Fig. 21B).

#### Habitat and ecology.

In wet, sub-Andine forests with *Oenocarpusbataua* Mart.(Arecaceae) at an elevation of ca. 1140 m. Flowering in November.

#### Etymology.

*Costusalfredoi* is named after the very active and inspiring Bolivian plant collector Alfredo Fuentes, who we personally met some years ago during a visit to the MO Herbarium and who provided us with additional data on Bolivian species of Costaceae.

#### Notes.

In Bolivia and Brazil, D. Skinner has observed plants with leaves and ligules that match this description. However, they were not in flower. The distribution of this species may be found to extend beyond the department of La Paz, Bolivia.

### ﻿ Costus alleniopsis

Taxon classificationPlantaeZingiberalesCostaceae

Maas & D.Skinner sp. nov.

09336D83-3917-5178-B7FE-6E7E4AC55868

urn:lsid:ipni.org:names:77316084-1

#### Diagnosis.

**Figure 3.**
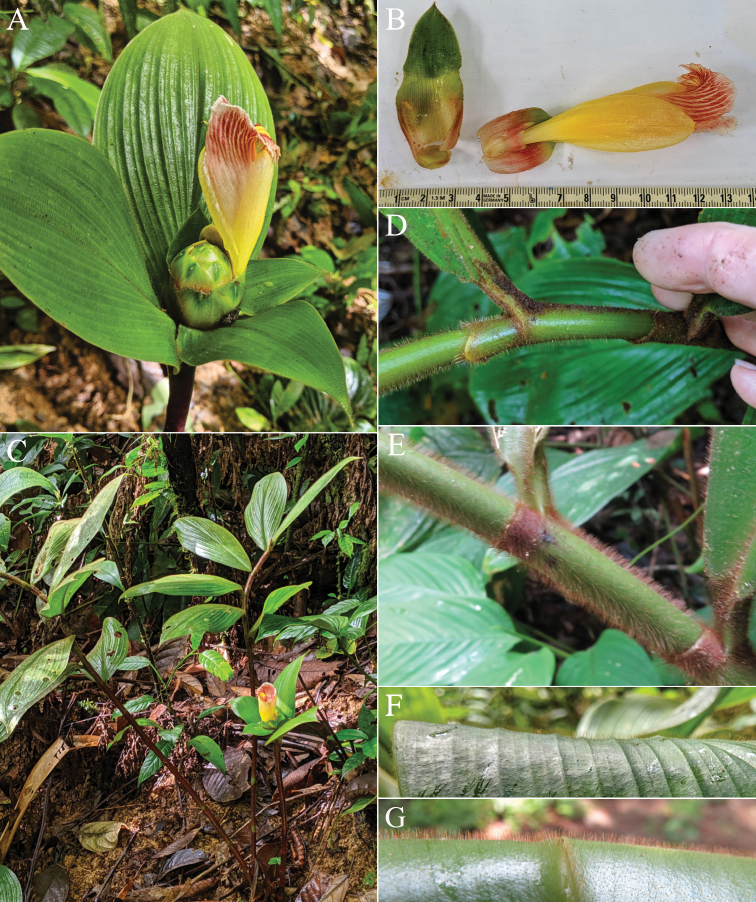
*Costusalleniopsis* Maas & D. Skinner **A** inflorescence **B** bracts and flower **C** plant in habitat **D** ligule and leaf base **E** ligule and leaf base of *C.allenii* for comparison **F** leaf plicae and indument **G** leaf plicae and indument of *Costusallenii* for comparison. Photos of *C.alleniopsis* in habitat, Veraguas, Panama by Dave Skinner.

*Costusalleniopsis* sp. nov. (Fig. 3) resembles *C.allenii* but differs by distinctly plicate leaves and a longer ligule.

#### Type.

Panama, Veraguas: Río Segundo Brazo, 700–750 m, 8 Sep 1974, *Maas & Dressler 1652* (holotype U1231561; isotypes F, MO-2193025).

#### Description.

***Herb*** 0.5–1.2 m tall. ***Leaves*** sheaths 10–20 mm diam; ligule obliquely truncate, 12–20 mm long, often reddish; petiole 10–25 mm long; sheaths, ligule and petiole densely brownish villose; lamina ovate to obovate, 25–32 by 13–16 cm, abaxially sometimes reddish, 10–12-plicate, adaxially and abaxially densely brownish villose, base cordate, acute, or obtuse, apex acuminate (acumen 10–15 mm long). ***Inflorescence*** ovoid, 6–7 × 3–5 cm, terminating a leafy shoot or rarely terminating a leafless shoot; bracts rather densely to densely brownish villose, bracteole, calyx, ovary, and capsule rather densely to densely puberulous to glabrous. ***Flowers*** adaxially oriented to erect; bracts green, rarely red, coriaceous, broadly ovate to ovate, 3–5 × 2–5 cm, apex obtuse, callus 5–7 mm long; bracteole boat-shaped, 20–30 mm long; calyx pink to red, 8–10 mm long, lobes shallowly triangular, 2–3 mm long; corolla yellow, 60–70 mm long, glabrous, lobes narrowly elliptic, 50–60 mm long; labellum yellow, distal edge horizontally spreading, broadly obovate, 50–70 × 40–50 mm, lateral lobes striped with red, middle lobe reflexed, with yellow honey mark, margin crenulate; stamen yellow, 40–50 × 12–15 mm, not exceeding the labellum, apex red, obtuse to irregularly dentate, anther 7–10 mm long. ***Capsule*** ellipsoid, 15–20 mm long.

#### Distribution.

Panama (Coclé, Colón, Veraguas) (Fig. 21C).

#### Habitat and ecology.

In forests, at elevations of 200–1300 m. Flowering in the rainy season.

#### Etymology.

This species strongly resembles *C.allenii*, and the Greek word “*opsis*” (= likeness) refers to the resemblance with that species.

#### Notes.

*Costusalleniopsis* sp. nov. is restricted to Panama. It is closest to *C.allenii* Maas, differing by a longer ligule (12–20 mm vs. 2–10 mm) and distinctly plicate leaves. They both share adaxial oriented to erect flowers, a feature not often seen in the genus *Costus*.

#### Paratypes.

**Panama. Coclé**: Alto Calvario, above El Copé, ca. 6 km N of El Copé, 23 Jun 1988, *Croat 68796* (MO, U); Alto Calvario, forest along Continental Divide, ca. 5 miles N of El Copé, 900–1000 m, 31 Mar 1993, *Croat 75052* (MO, U); Caribbean side of divide at El Copé, 200–400 m, 4 Feb 1983, *Hamilton & Davidse 2778* (MO); near sawmill, 8 km N of El Copé (28 km NW of Penonomé), 600–750 m, 1 Sep 1977, *Maas et al. 2760* (U); Parque Nacional General Omar Torrijos Herrera, El Copé, Sendero Rana Dorada, 750 m, 31 May 2004, *Maas et al. 9541* (U); above El Potroso sawmill at Continental Divide, N of El Copé, 1200–1300 m, 13 May 1981, *Sytsma & Andersson 4610* (MO, U). **Colón**: MPSA Concession, around Petaquilla Tower, 23 May 2012, *Hammel et al. 26342* (MO). **Veraguas**: valley of Río Dos Bocas, on road between Alto Piedra (above Santa Fé) and Calovebora, 350–400 m, 29 Aug 1974, *Croat 27455* (MO, U); near the Continental Divide, ca. 15 km NW of Santa Fé, 800 m, 22 Oct 1980, *Maas & Dressler 5006 p.p.* (K, L, U); 16 km NW of Santa Fé, on road to Calovebora (Panamanian Highway 35), 300–500 m, 16 May 1975, *Mori & Kallunki 6124* (MO).

### ﻿ Costus alticolus

Taxon classificationPlantaeZingiberalesCostaceae

Maas & H.Maas sp. nov.

5B398AFC-E2A9-5824-B19C-58E6A19DE6EB

urn:lsid:ipni.org:names:77316085-1

#### Diagnosis.

**Figure 4.**
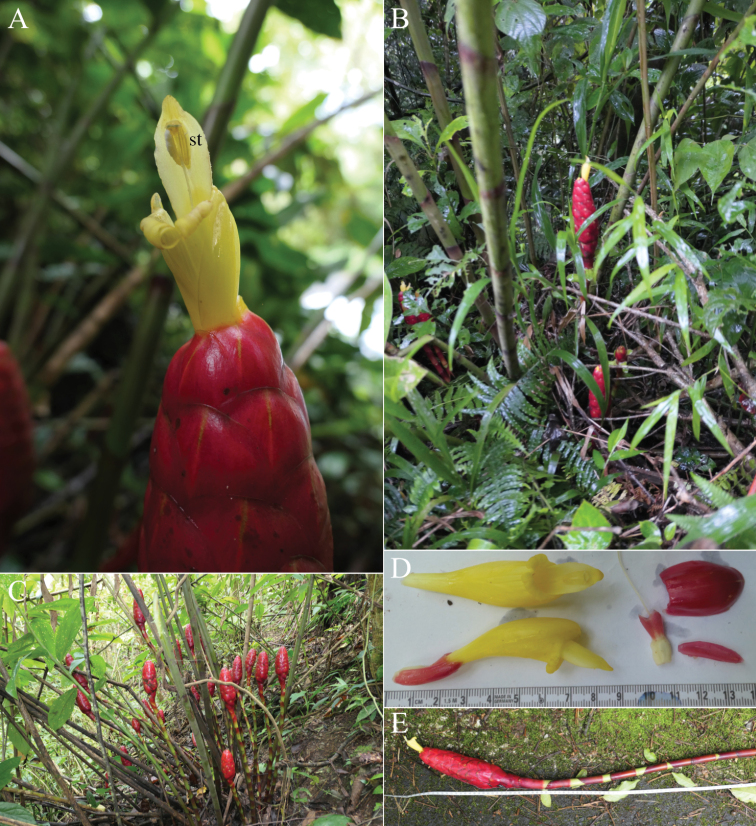
*Costusalticolus* Maas & H.Maas **A** inflorescence with flower, showing stamen (st) extending far beyond labellum, fully exposing the thecae **B, C** plant in habitat **D** flower and bract details **E** flowering shoot. Photos in habitat in Chinantla region near Santa Cruz Tepetotutla, Oaxaca, Mexico by Dave Skinner.

*Costusalticolus* sp. nov. (Fig. 4) can be recognised by its leafless flowering shoots up to ca. 1 m long, combined with large, red and unappendaged bracts, tubular and yellow flowers, and stamen far exceeding the labellum like in *C.pulverulentus* C.Presl.

#### Type.

Mexico. Oaxaca: Sierra Mazateca, Mun. Santa María Chilchotla, Cuauhtemoc, ca. 4 km NE of Santa María Chilchotla, near Clemencia and Santa Rosa, 1200 m, 16 Jan 1984, *Solheim & Reisfeld 1367* (holotype WIS).

#### Description.

***Herb*** 2–3.5 m tall. ***Leaves*** sheaths 10–30 mm diam; ligule truncate, 4–12 mm long; petiole 5–20 mm long; sheaths, ligule and petiole densely to sparsely puberulous; lamina narrowly elliptic, 25–47 × 6–14 cm, adaxially glabrous, abaxially densely (particularly along the primary vein) sericeous to almost glabrous, base acute, apex acuminate (acumen 5–10 mm long). ***Inflorescence*** cylindric, 11–18 × 5–9 cm, terminating a leafless shoot 20–95 cm long, sheaths obliquely truncate, 4–7 cm long, rather densely puberulous to glabrous; bracts, bracteole, calyx, ovary, and capsule glabrous to densely puberulous. ***Flowers*** abaxially oriented; bracts red to bright crimson, coriaceous, ovate-oblong, 4–6 × 3–4 cm, apex obtuse, callus ca. 10 mm long; bracteole boat-shaped, 30–35 mm long; calyx red, 13–21 mm long, lobes deltate to shallowly triangular, 3–6 mm long; corolla yellow to orange, 50–60 mm long, glabrous, lobes narrowly elliptic, ca. 40 mm long; labellum yellow, lateral lobes rolled inwards and forming a slightly curved tube 18–20 mm diam, obovate when spread out, ca. 60 by 35 mm, irregularly lobulate, lobules recurved, ca. 4 mm long; stamen yellow, ca. 65 × 11 mm, far exceeding the labellum, apex acute, anther ca. 8 mm long. ***Capsule*** ellipsoid, 17–18 mm long.

#### Distribution.

Mexico (Oaxaca) (Fig. 21D).

#### Habitat and ecology.

In primary, wet montane to premontane cloud forests, with some mosses on trunks, drier on ridge crests, with *Hedyosmummexicanum* Cordem. Ex Baill., *Liquidambarmacrophylla* Oerst., *Siparunaandina* A.DC., *Posoquerialatifolia* (Rudge) Roem. & Schult., *Heliconia* sp., *Deppea* sp, *Chamaedorea* sp., *Solenophora* sp., and *Salviadivinorum* Epling & Játiva, at elevations of 1040–1900 m. Flowering year-round.

#### Etymology.

*Costusalticolus* sp. nov. occurs at quite high elevations up to 1900 m, hence the specific epithet alticolus (‘altus’ means ‘high’ in Latin; ‘colere’ means ‘to live’ in Latin).

#### Paratypes.

**Mexico. Oaxaca**: Distr. and Mun. Ixtlán, near abandoned community of Tarabundi, S side of Río Soyolapán, access via La Luz, primary forest toward Río Langaro, 1230–1260 m, 16 Nov 1993, *Boyle & Massart 2556* (MO, U); Mun. San Felipe Usila, cuenca del Río Perfume, 4.4 km SE of Santa Cruz Tepetotutla, 1400 m, 29 Mar 1995, *Rincón Guttiérrez et al. 592* (MO); Distr. Mixe, Mun. Totontepec, Totontepec, 1900 m, 9 Aug 1990, *Rivera Reyes & Martin 1610* (CAS), Hwy 175, km 169, near La Esperanza, 1075 m, 12 Aug 2017, *Skinner R3400* (BH).

#### Notes.

We are greatly indebted to Manuel Gutiérrez, who assisted Dave Skinner in obtaining permission from the indigenous people of Santa Cruz Tepetotutla, Oaxaca, Mexico, to study this new species common in the Chinantla region of Oaxaca.

### ﻿ Costus antioquiensis

Taxon classificationPlantaeZingiberalesCostaceae

Maas & H.Maas sp. nov.

6C2E3952-1D7D-51B4-9108-20D0CCE6FA23

urn:lsid:ipni.org:names:77316086-1

#### Diagnosis.

**Figure 5.**
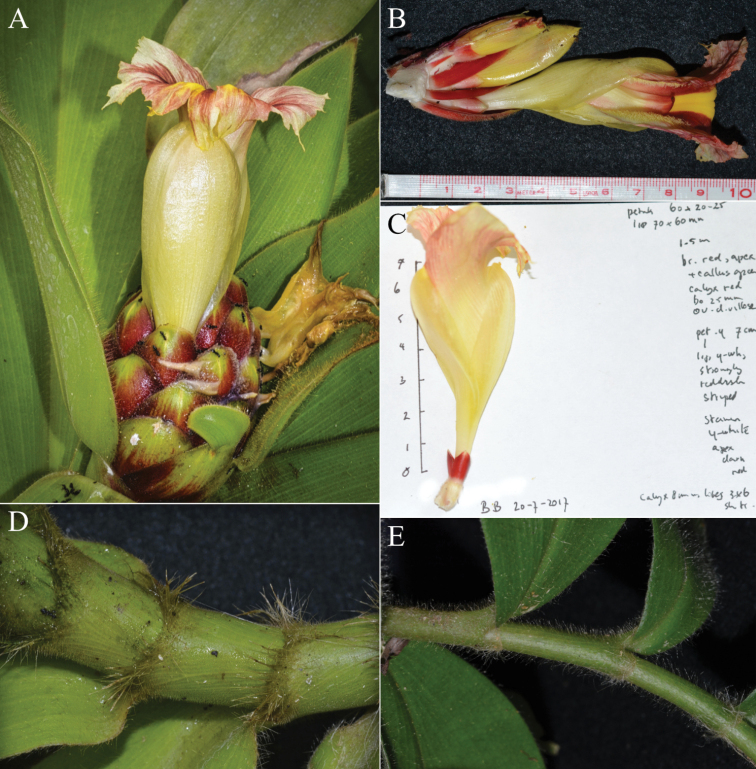
*Costusantioquiensis* Maas & H.Maas **A** inflorescence **B** flower **C** flower details and handwritten notes by Paul Maas **D** detail of hairy ligule **E** sheath. Photos **A–E** of specimen *Maas et al 10488* taken prior to pressing. Photos by Paul Maas.

**Figure 6.**
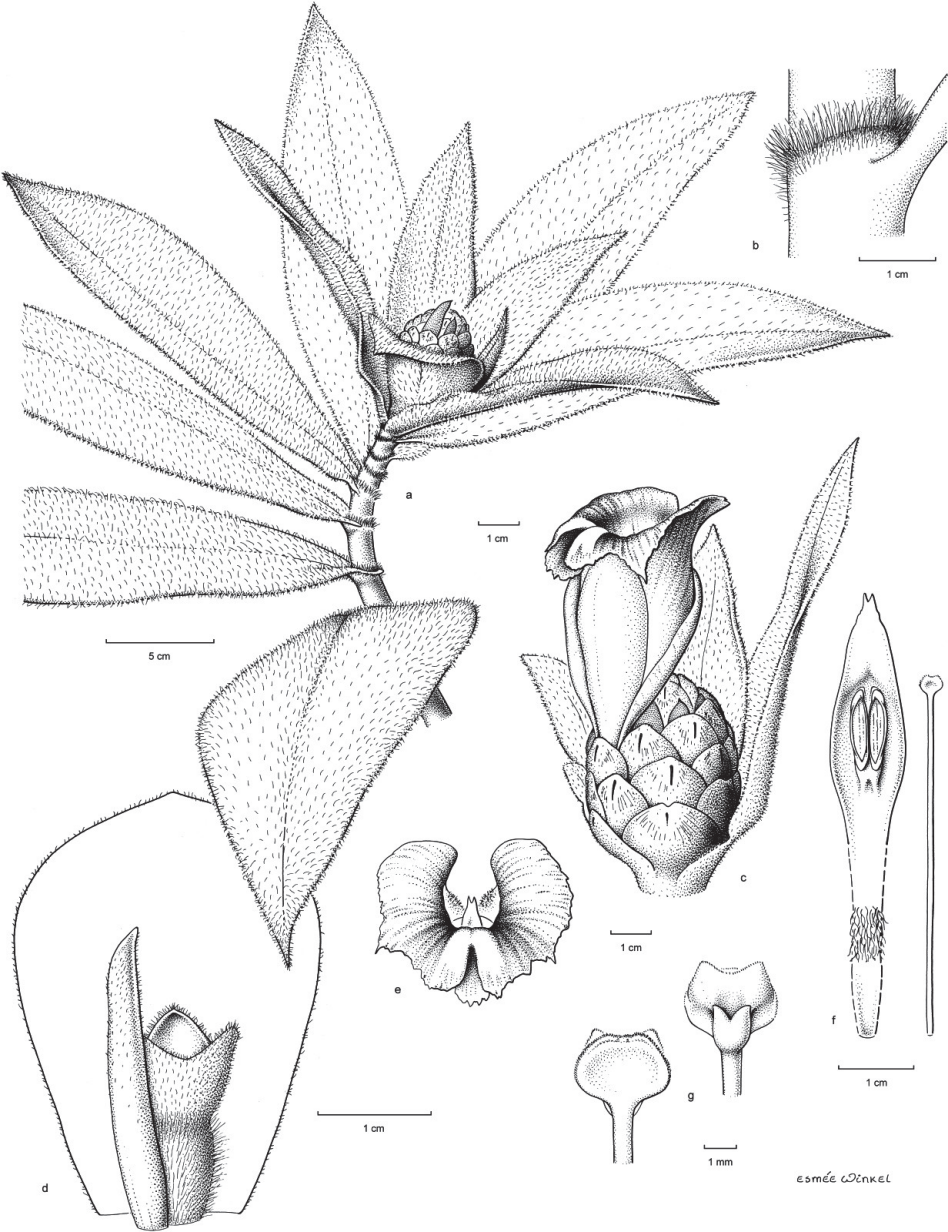
*Costusantioquiensis* Maas & H.Maas **A** inflorescence terminating a leafy shoot **B** ligule showing indument at margin **C** inflorescence close up with flower at anthesis **D** bract (adaxial surface) subtending a bracteole (left), calyx with three lobes, and ovary at the base of the calyx **E** labellum as viewed from front, with the forked tip of the single fertile stamen visible at the center **F** stamen (left) and style (right) **G** stigma close-up, dorsal (left) and ventral (right) views. The forked projection on the ventral surface hooks between the thecae of the fertile stamen to hold the style erect. Drawing by Esmée Winkel.

*Costusantioquiensis* sp. nov. (Figs 5, 6) is well recognisable by adaxially oriented flowers, an often present ring of brown, erect and rather stiff hairs at the base of the ligule, and the abaxial leaf lamina and the bracts being often densely villose. It could be confused with *Costuslaevis* Ruiz & Pav., but that species has much darker flowers and is often completely glabrous.

#### Type.

Colombia, Antioquia: Mun. San Rafael, along road from Guatapé to San Rafael, Río El Bizcocho, Finca El Castillo, 1059 m, 12 Oct 2013, *Maas*, *Maas-van de Kamer & Valderrama 10488* (holotype L, 3 sheets; isotypes JAUM, K, MO-3159234, MO-3159235).

#### Description.

***Herb*** 0.5–5 m tall. ***Leaves*** sheaths 10–20 mm diam; ligule truncate, 5–10 mm long, basally densely covered with a ring of brown, erect and rather stiff hairs (villose), sometimes absent; petiole 5–20 mm long; sheaths and petiole sparsely or sometimes densely villose; lamina narrowly elliptic, 30–50 × 9–13 cm, adaxially glabrous, sometimes densely villose, abaxially densely to sometimes sparsely villose or glabrous, base obtuse, apex acuminate (acumen 5–10 mm long). ***Inflorescence*** ovoid, 8–16 × 5–6 cm, enlarging to ca. 25 by 7–8 cm in fruit, terminating a leafy shoot; bracts, bracteoles, calyx, ovary, and capsule densely puberulous to villose, rarely glabrous. ***Flowers*** adaxially oriented to erect; bracts green, coriaceous, broadly ovate, 3–4 × 3–4 cm, apex obtuse, callus 8–10 mm long; appendages rarely present; bracteole boat-shaped, 20–27 mm long; calyx (pale) red, 7–11 mm long, lobes deltate to shallowly triangular, ca. 3 mm long; corolla yellow, pale orange, or pink, 60–70 mm long, glabrous, lobes elliptic, 40–60 mm long; labellum yellowish white, distal edge horizontally spreading, broadly obovate, 50–70 × 40–60 mm, lateral lobes striped with red, middle lobe reflexed, with dark yellow honey mark, margin irregularly dentate; stamen yellowish white to pink, ca. 40 × 10–12 mm, not exceeding the labellum, apex dark red, entire to dentate, anther ca. 8 mm long. ***Capsule*** obovoid, 25–28 mm long.

#### Distribution.

Colombia (Amazonas, Antioquia, Arauca, Boyacá, Caldas, Cauca, Chocó, Córdoba, Cundinamarca, Guajira, Guaviare, Huila, Meta, Norte de Santander, Quindío, Risaralda, Santander, Tolima, Valle del Cauca, Vichada), Venezuela (Mérida), Ecuador (Azuay, Carchi, Pastaza, Zamora-Chinchipe), Peru (Ucayali). (Fig. 21E)

#### Habitat and ecology.

In forests (a.o., “selva baja/alta perennifolia” or wet montane forests) or forested roadsides at elevations of 50–1700 m. Flowering year-round.

#### Vernacular names.

Colombia: Caña agria, Cañagria, Cañaguate. Peru: Sacha huiro.

#### Etymology.

This species was collected by Paul and Hiltje Maas for the first time in the Colombian department of Antioquia and is therefore named in recognition of that locality.

#### Paratypes.

**Colombia. Amazonas**: Mun. Puerto Nariño, Trapécio Amazónico, orillas del Amazonas, 1 Nov 1945, *Duque-Jaramillo 2251* (COL). **Antioquia**: Mun. Frontino, Corregimiento Nutibara-La Blanquita, 4–7.1 km between Alto de Cuevas and La Blanquita, 1420–1610 m, 26 Jan 1995, *Betancur B. et al. 5970* (COL, U, US); Carauta, Mun. Frontino, Corregimiento Carauta, Parque Nacional Natural “Las Orquídeas”, sector Tres Bocas, quebrada San Francisco, poco antes de la desembocadura al río Carauta, Finca La Pradera, 1640–1680 m, 3 Sep 2012, *Betancur B. et al. 16507* (NY); Mun. Frontino, Corregimiento Carauta, Parque Nacional Natural “Las Orquídeas”, sector Tres Bocas, margen derecha del río Carauta, poco después de Tres Bocas, Finca La Pradera, 1610 m, 12 Sep 2012, *Betancur B. et al. 16651* (NY). **Arauca**: Campo petrolero de Caño Limón, Consorcio Cravo Norte, cerca del relleno sanitario, 280 m, 16 Oct 1997, *Betancur B. 7500* (COL). **Boyacá**: Puerto Boyacá, Corregimiento Puerto Romero, quebrada alrededores del campamento Techint, 10 Oct 1996, *Balcázar 49* (COL); Guacamayas, Vereda La Laguna, sector Litargón, 2349 m, 7 Jun 2009, *Beltrán Cuartas 78* (COL). **Caldas**: Mun. Norcasia, Reserva Natural Ríomanso, ca. 40 km de La Dorada, valle del río Magdalena, 212 m, 14 Oct 2013, *Guevara-Ibarra 39* (COL, CUVC); Mun. Victoria, Vereda Gigante, Hacienda La Española, 314–321 m, 5 Jun 2014, *Vargas-Figueroa et al. 855* (CUVC). **Cauca**: Mun. Santander del Quillichao, Hacienda Nana Loisa, 1000 m, 2 Nov 2002, *Silverstone-Sopkin et al. 8963* (CUVC). **Chocó**: Carretera Tutunendo to El Carmén, entre kms 135 y 120, 800–1200 m, 29 Apr 1979, *Forero et al. 6161* (COL, MO, U); Mun. San José del Palmar, mouth of Río Torito (affluent of Río Habita), 850–950 m, 16 May 1980, *Forero et al. 7413* (COL, U). **Córdoba**: Montería, Buenos Aires, Finca La Poderoza, 13 Apr 2005, *Pulido 107* (COL). **Cundinamarca**: Yacopí, Inspección de policía de Guadualito, Vereda del Lamal, al lo largo de la carretera de El Lamal a El Gramal, 950–1215 m, 26 Oct 1998, *Galeano et al. 2036* (COL); Yacopí, Churupaco, areas llamadas Peladeros, 1700 m, 8 Feb 1956, *Idrobo 2027* (COL). **Guajira**: 16 km S of Carraipia, Corregimiento Guajira, 400 m, 6 Aug 1944, *Haught 4292* (MEDEL, US). **Guaviare**: Mun. San José del Guaviare, Vereda Agua Bonita, Finca El Caimám, 1178 m, 17 Apr 2008, *Vélez et al. 7073* (HUA). **Huila**: Cordillera Oriental, Vertiente occidental, montes más arriba de Guadelupe, 1600–1700 m, 20 Mar 1940, *Cuatrecasas & Pérez-Arbeláez 8396* (COL, F); Neiva, Vereda Tamarindo, Finca La Tribuna, camino al pozo San Francisco 67, 550 m, 18 Apr 2004, *Obando 284* (COL). **Meta**: Carretera Buenavista-Villavicencio, *Duque-Jaramillo 3912 A* (COL, NY); Villavicencio, Reserva Bavaria. 1000 m, 15 Nov 1995, *Lozano-Contreras 7397* (COL). **Norte de Santander**: Hoya del río Sardinata, Finca La Soledad y carretera a Tibú, 50–70 m, 10 May 1965, *García-Barriga & Lozano-Contreras 18240* (COL). **Quindío**: Mun. Montenegro, Vereda El Gigante, Finca El Porvenir, ladera del río Roble, 1100–1200 m, 2 May 2003, *Cordero-P. et al. 91* (COL). **Risaralda**: Mun. Pereira, Hacienda La Alejandria, en la vía Cerritos-La Virginia, 900 m, 24 Jan 1991, *Ramos-Pérez et al. 3052* (CUVC, U); Mun. Pereira, Hacienda Alejandria, km 6 of road Cerritos-La Virginia, 900 m, 1 Dec 1989, *Silverstone-Sopkin et al. 5811* (CUVC, U). **Santander**: Puerto Parra-Campo Capote, 24 Jan 1991, *Rentería Arriaga et al. 2123* (COL, HUA, JAUM, MO). **Tolima**: Melgar, Río Sumapán, Hacienda Corinto, 350 m, 26 Sep 1974, *Maas & Jaramillo Mejía 1783* (COL); Ibagué, Hacienda Altamira (Laserna), 5 Apr 2014, *Villanueva & Cabezas 1844* (COL). **Valle del Cauca**: Cordillera Occidental, hoya del río Digua, Quebrada del Cauchal, 300 m, 19 Dec 1942, *Cuatrecasas 13714* (F, US); Cordillera Central, near Palmira, 1400 m, 3 Jan 1972, *Maas & Escobar 596* (U); Quebrada Nueva to Cuchilla, E of Zarzal, 1100–1300 m, 21 Jul 1922, *Pennell et al. 8486* (NY, US); Mun. Zarzal, Hacienda El Medio (Carretera Panamericana between La Paila and Zarzal), 950 m, 15 Apr 1987, *Silverstone-Sopkin et al. 3085* (CUVC, U). **Vichada**: Cumaribo, Cerro Thomas, 50 m, 26 Aug 2006, *Rojas-Zamora 136* (COL). **Ecuador. Azuay**: Highway Cuenca-Cola de San Pablo, Contego encampment, 1640 m, 15 Feb 1977, *Boeke & Loyola 1009* (L). **Carchi**: Peñas Blancas, 20 km below Maldonado, on the Río San Juan, 900–1000 m, 27 May 1978, *Madison et al. 4623* (F, QCA, SEL, U). **Pastaza**: valley of the Río Pastaza, near El Topo, 4000–5000 ft, 17 Apr 1945, *Camp E 2395* (NY, US). **Zamora-Chinchipe**: along road from Namirez to Nambija, just S of Nambija, 1779 m, 23 Jul 2004, *Croat 92034* (MO, QCNE, U). **Peru. Ucayali**: Prov. Coronel Portillo, Distr. Padre Abad, La Divisoria, near Río Chino, 1500–1600 m, 3 Jun 1978, *Schunke V. 10195* (MO, U). **Venezuela. Mérida**: Carretera Panamericana, 15 km SW of El Vigia, 50–130 m, 6 Jun 1952, *Vareschi & Pannier 1674* (M), 4 Mar 1968, *Wessels Boer et al. 2440* (U). **Cultivated Material** Cultivated in Burgers’ Bush, Arnhem, the Netherlands, from *Maas et al. 10488* from Colombia, *P.J.M. & H. Maas 10631* (L).

#### Notes.

*Costusantioquiensis* sp. nov. could be confused with *C.allenii* Maas, but in that species, the entire surface of sheaths, ligule and petiole are villose, while *C.antioquiensis* is highly variable in its indument. Most material has a quite dense villose indument all over the plant, but some specimens of the same population can be almost completely glabrous, especially the adaxial side of the leaves. The ring of erect hairs at the base of the ligule is almost always present in *C.antioquiensis* but consistently lacking in *C.allenii*.

*C.antioquiensis* sp. nov. also looks similar to *C.laevis* Ruiz & Pav. and was misidentified by us as belonging to that species in the past. It shares with *C.laevis* the adaxially oriented flowers, but the flowers are a much lighter colour in *C.antioquiensis*. Moreover, there are notable differences in indumentum, which is almost completely lacking in *C.laevis* Ruiz & Pav., whereas, in *C.antioquiensis*, the vegetative parts are mostly densely villose.

### ﻿ Costus callosus

Taxon classificationPlantaeZingiberalesCostaceae

Maas & H.Maas sp. nov.

A7EA1648-C6B8-5EB6-8D92-DD79EA7064BF

urn:lsid:ipni.org:names:77316087-1

#### Diagnosis.

**Figure 7.**
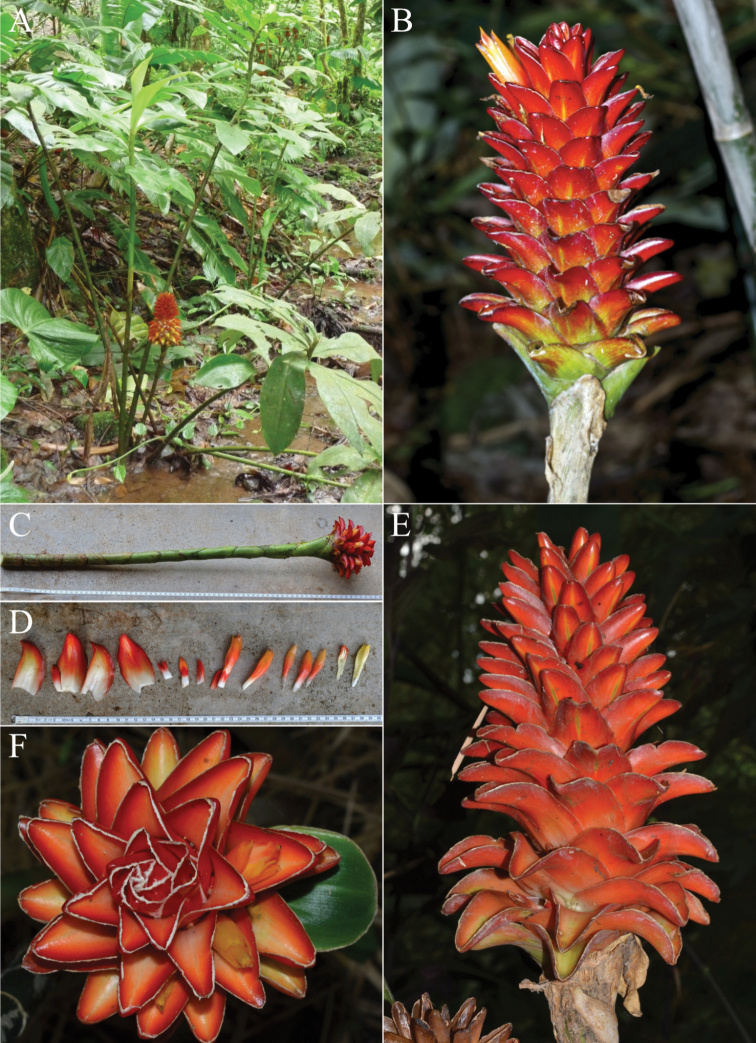
*Costuscallosus* Maas & H.Maas **A** plant in habitat **B** inflorescence **C** separate flowering shoot **D** bract and flower details showing very long bract callus **E, F** cultivated plant **A, C, D** observation from Willie Mazu site, Bocas del Toro, Panama **B** plant in cultivation at Lyon Arboretum 2003.0125, collected in Costa Rica by Alan Carle **E, F** plant observed at Reserva Natural Nirvana, Valle del Cauca, Colombia. Photos **A, C, D** by Dave Skinner, **B** by John Mood, **E, F** by Paul Maas.

*Costuscallosus* sp. nov. (Fig. 7) is most similar to *C.curvibracteatus*, differing by the inflorescence mostly produced terminally on a leafless shoot, with soon withering sheaths and bracts with a very long callus.

#### Type.

Colombia, Chocó: Mun. San José del Palmar, mouth of Río Torito (affluent of Río Hábita), Finca Los Guaduales, 730–830 m, 4 Mar 1980, *E. Forero*, *Jaramillo-Mejía*, *Espina Z. & Palacios 6668* (holotype COL, 3 sheets; isotypes MO-1456278, U1607067, U1607068, U1607069, U1610613).

#### Description.

***Herb*** 0.5–3 m tall. ***Leaves*** sheaths 15–30 mm diam; ligule obliquely truncate, 10–30 mm long, distally black to dark purple, with a minute, salient rim; petiole 5–20 mm long; sheaths, ligule and petiole glabrous, sparsely puberulous, or densely villose; lamina narrowly elliptic, 25–50 × 7–16 cm, adaxially sparsely to rather densely villose to puberulous, abaxially rather densely villose, base acute, apex acuminate (acumen 5–15 mm long). ***Inflorescence*** ovoid to subglobose, 6–23 × 6–12 cm, terminating a leafless shoot 20–90 cm long, rarely terminating a leafy shoot (*Knapp & Vodicka 5524*), sheaths withering and completely or partly falling off with age, obliquely truncate, 4–10 cm long, glabrous, sparsely puberulous to densely villose; bracts, bracteole, calyx, ovary, and capsule glabrous to rather densely puberulous. ***Flowers*** abaxially oriented; bracts orange-red, red, or yellow, coriaceous, ovate-triangular, 4–7 × 2.5–4 cm, apex acute to obtuse, more or less curved outwards to patent in living plants, callus 8–25 mm long, margins wooly in living material; bracteole boat-shaped, 20–30 mm long; calyx red, 10–18 mm long, lobes deltate, 2–8 mm long; corolla orange to yellow, ca. 50 mm long, glabrous, lobes elliptic, ca. 30 mm long; labellum yellow, lateral lobes rolled inwards and forming a tube ca. 10 mm diam, elliptic when spread out, ca. 35 × 25 mm, 3-lobulate, lobules red, 5–6 mm long; stamen yellow, 35–40 × 12–15 mm, slightly exceeding the labellum, apex orange-red, acute, anther 9–10 mm long. ***Capsule*** ellipsoid to obovoid, 8–15 mm long.

#### Distribution.

Costa Rica, Panama, Colombia (Antioquia, Chocó) (Fig. 21F).

#### Habitat and ecology.

In forests, at elevations of 0–1300 m. Flowering year-round.

#### Vernacular names.

Panama: Caña agria (Kuna).

#### Etymology.

*Costuscallosus* sp. nov. has been named after the very conspicuous thickened callose zone on its bracts.

#### Paratypes.

**Costa Rica. Alajuela**: Volcán Arenal, *Funk 10490* (MO). **Heredia**: path beyond Río Sucio, Braulio Carrillo, 400 m, 4 May 1984, *Gómez Pignataro et al. 21201* (MO); Cantón de Sarapiquí, Llanura de Tortuguero, ca. 5 km NE of Puerto Viejo, NE side of Río Sucio, 50 m, 5 Oct 1996, *Hammel et al. 20494* (INBIO, MO, U, 2 sheets); Cantón de Sarapiquí, Cuenca del Sarapiquí, 5 km NE of Puerto Viejo, NE side of Río Sucio, 50 m, 22 Sep 1996, *Hammel et al. 20694* (INBIO, MO). **Limón**: Hacienda Tapezco-Hacienda La Suerte, 29 air km W of Tortuguero, 40 m, 24 Aug 1979, *Davidson & Donahue 8770* (CR, LAM, U); Parque Nacional Braulio Carrillo, W side of Río Sucio, 450 m, 4 Aug 1989, *Hammel et al. 17668* (MO, U); Braulio Carrillo National Park, Quebrada Gonzales section, ca. 40 km NNE of San José, 500 m, May 1992, *Lücking s.n.* (U). **Panama. Bocas del Toro**: 2 miles below the divide along main highway, 500 m, 23 Jun 1986, *Kress et al. 86-1978* (MO). **Chiriquí**: behind Vivero Forestal de Boquete, 12 km N of Los Planes de Hornito, IRHE Fortuna, 1100–1200 m, 17 Jun 1982, *Knapp & Vodicka 5524* (U); near Fortuna dam, from Continental Divide to Chiriquí Grande, 1100 m, 22 Oct 1985, *LaFrankie 85-38* (MO, U). **Darién**: Parque Nacional del Darién, along S branch of Río Pucuro, near Tacarcuna, ca. 18 km E of Pucuro, 600–800 m, 25 Oct 1987, *Hammel et al. 16513* (MO, U); 0–2 miles E of Tres Bocas, along the shortest headwater of Río Cuasi, 28 Apr 1968, *Kirkbride & Duke 1180* (MO, U). **Panama**: Altos de Pacora, ca. 20 km NE of Cerro Azul, 750 m, 19 May 1972, *Dressler 4190* (U, 3 sheets); summit of Cerro Jefe, 26 Aug 1967, *Hayden 1020* (MO, 2 sheets); 5–6 hours walk from Chocó village, Serrania de Maje, 650–800 m, 31 Mar 1982, *Knapp et al. 4519* (MO, U); Altos de Pacora, 750 m, 2 Sep 1974, *Maas et al. 1548* (U, 2 sheets). **Colombia. Antioquia**: Mun. Amalfi, between Amalfi and Fraguas, 1220–1300 m, 14 Feb 1989, *MacDougal et al. 4020* (HUA). **Chocó**: Carretera Panamericana, between Río San Pablo and Río Pató, 23 Apr 1979, *Forero et al. 5765* (COL, MO, U, 2 sheets). **Cultivated Material.** Colombia, Valle del Cauca, Mun. Palmira, Reserva Natural Nirvana, 13 Jul 2018, *Maas et al. 10688* (L, 3 sheets).

#### Notes.

Maas (1977) already suspected this species differed from *C.curvibracteatus* Maas, although they share many ligule and bract characters. The presence of a separate, leafless flowering shoot with soon withering sheaths and the presence of a distinct callus on the bracts convinced us that it is worthy of recognition as a separate species. Material recently collected (*Maas et al. 10688*) in the Reserva Natural Nirvana, Valle del Cauca, Colombia, made it possible to add all flower details to the description.

### ﻿ Costus cochabambae

Taxon classificationPlantaeZingiberalesCostaceae

Maas & H.Maas sp. nov.

613A10E7-BCF6-580E-A416-6906D9BB532A

urn:lsid:ipni.org:names:77316088-1

#### Diagnosis.

**Figure 8.**
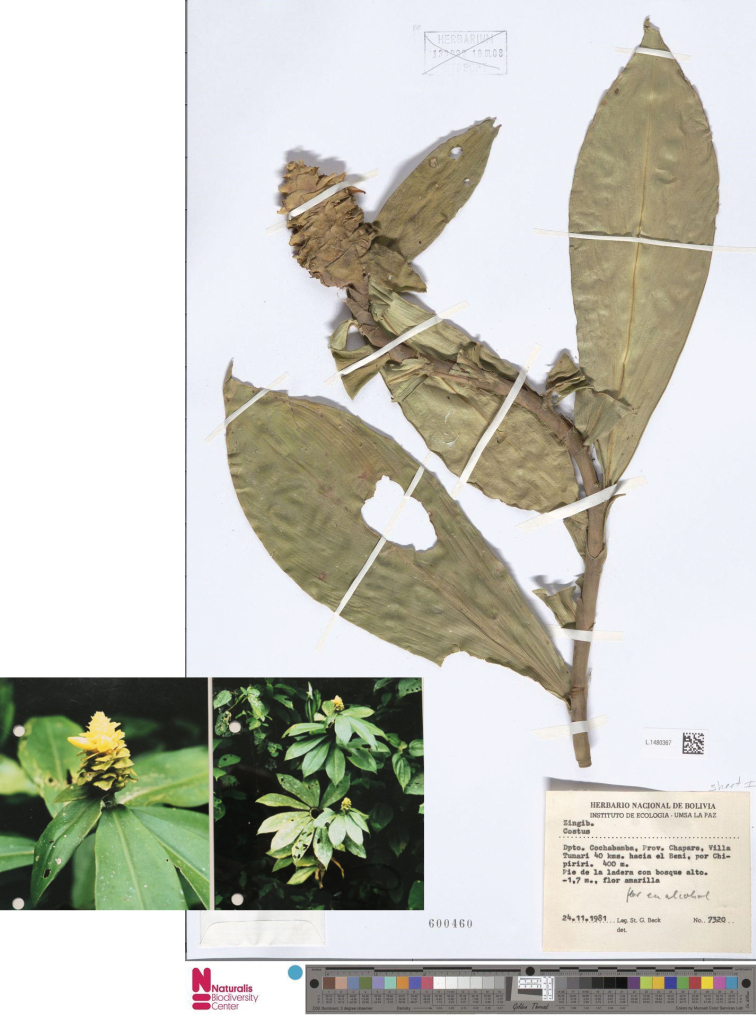
*Costuscochabambae* Maas & H.Maas Isotype photographed at Leiden, L.1480367 including the photos enclosed with the specimen, *Beck 7320*.

**Figure 9.**
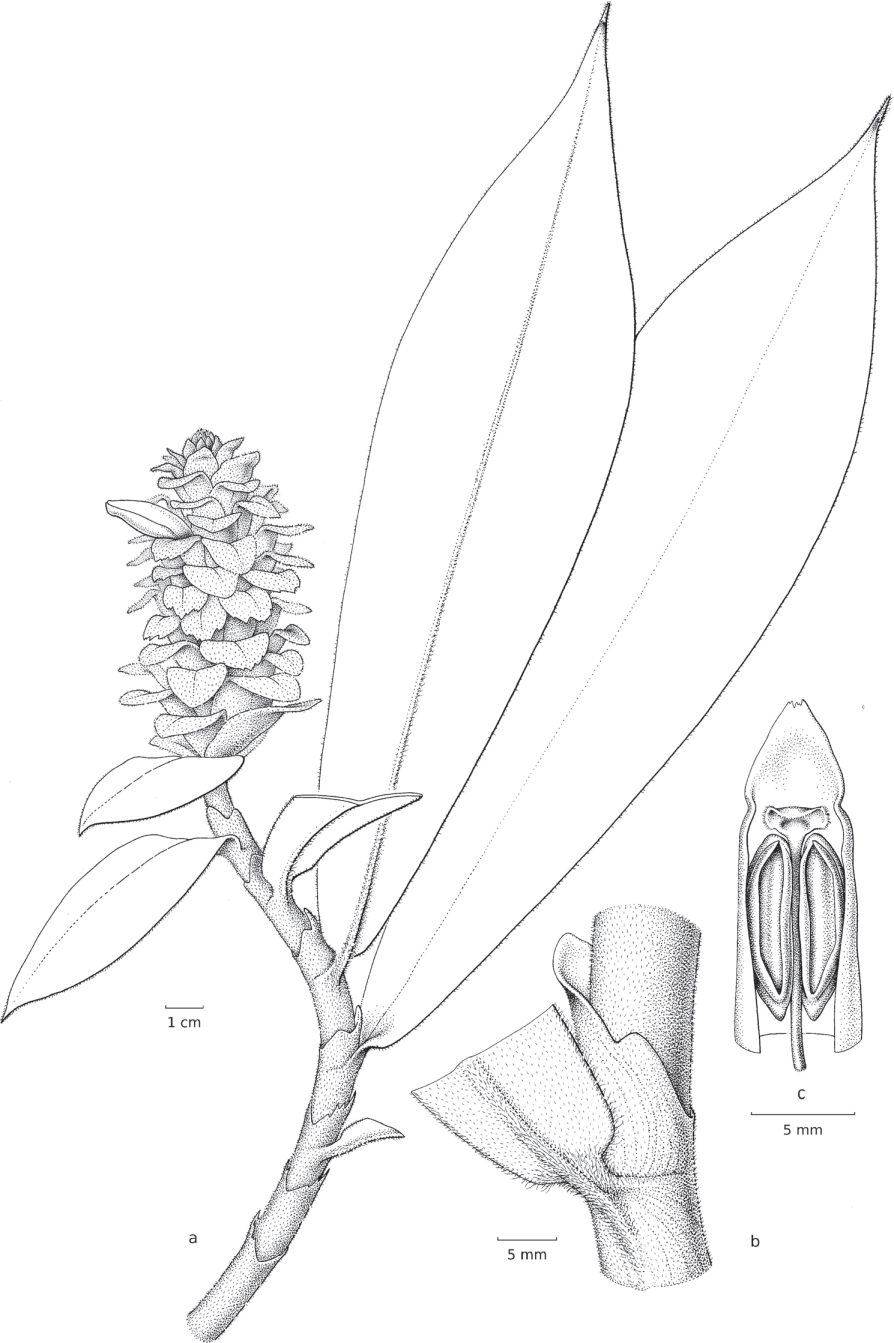
*Costuscochabambae* Maas & H.Maas **A** habit **B** leaf base with ligule **C** stamen. Drawing by Esmée Winkel.

*Costuscochabambae* sp. nov. (Figs 8, 9) superficially resembles *C.comosus* but differs by greenish appendaged bracts, a subglabrous corolla, a glabrous lower leaf side (except for the hairy primary vein), and a much longer ligule up to 25 mm long (vs. 1–3 mm).

#### Type.

Bolivia, Cochabamba: Prov. Chapare, Villa Tunari, 40 km hacia el Beni, por Chipiriri, 400 m, 24 Nov 1981, *Beck 7320* (holotype LPB, specimen lacking a barcode; isotypes L, 2 sheets: L1480367 & L1480368 and spirit collection: L0303088).

#### Description.

***Herb*** ca. 1.7 m tall. ***Leaves*** sheaths 5–8 mm diam; ligule 15–25 mm long, unequally lobed, lobes obtuse; petiole ca. 5 mm long; sheaths, ligule and petiole densely to rather densely puberulous to villose; lamina narrowly elliptic to narrowly obovate, 17–30 × 6–8 cm, adaxially glabrous, abaxially glabrous with primary vein densely villose, strongly raised and prolonged as a prominent rim into the sheaths, base obtuse, apex acuminate (acumen 5–10 mm long). ***Inflorescence*** ovoid, 5–8 × 3–5 cm, terminating a leafy shoot; bracts, bracteole, and calyx densely to rather densely puberulous, ovary and capsule densely puberulo-sericeous. ***Flowers*** abaxially oriented; bracts green, coriaceous, broadly ovate, 1.5–2 × 1.5–2 cm; appendages green, foliaceous, reflexed when dry, broadly to shallowly ovate-triangular, 0.5–1.2 × 1.2–2 cm, apex obtuse to acute; bracteole boat-shaped, 15–17 mm long; calyx 9–12 mm long, lobes shallowly ovate-triangular, 2–3 mm long; corolla yellow, 30–40 mm long, subglabrous, lobes elliptic, 20–25 mm long; labellum yellow, lateral lobes rolled inwards and forming a tube ca. 10 mm diam, oblong-elliptic when spread out, ca. 20 × 15 mm, dentate; stamen yellow, ca. 20 × 5 mm, not exceeding the labellum, apex dentate, anther 8–9 mm long. ***Capsule*** not seen.

#### Distribution.

Bolivia (Cochabamba) (Fig. 21G).

#### Habitat and ecology.

In high forests (“pie de la ladera con bosque alto”), at an elevation of ca. 400 m. Flowering in November.

#### Etymology.

This species is named after the Bolivian department of Cochabamba, where it was collected.

#### Notes.

*Costuscochabambae* sp. nov. resembles *C.comosus* and is likely to be closely related to this species.

### ﻿ Costus convexus

Taxon classificationPlantaeZingiberalesCostaceae

Maas & D.Skinner sp. nov.

76FE595F-BCE6-5E86-89C9-9D7A88B3B36F

urn:lsid:ipni.org:names:77316089-1

#### Diagnosis.

**Figure 10.**
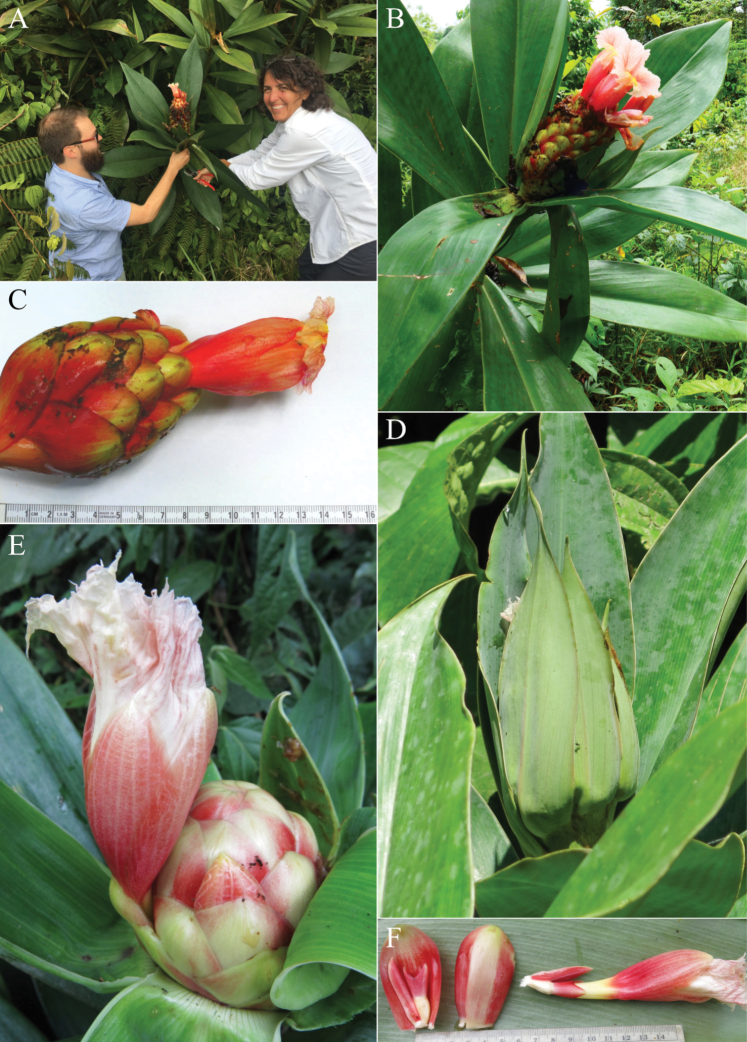
*Costusconvexus* Maas & D.Skinner **A** plant in habitat with Eugenio Valderrama (left) and Chelsea Specht (right) **B** inflorescence **C** inflorescence with scale **D** upper leaves wrapping around and hiding the inflorescence **E** close-up of inflorescence **F** bract (left) and flower (right) detail. Photo in **A** taken in the wild prior to pressing *Maas 10707*, Putumayo, Colombia **B, C** plant observed in Putumayo, Colombia **D–F** plant observed in Zamora-Chinchipe, Ecuador. Photo **A** by Paul Maas **B–F** by Dave Skinner.

*Costusconvexus* sp. nov. (Fig. 10) shares with *C.glaucus* Maas the presence of glaucous shoots and leaves especially in young plants, but differs from that species by a globose inflorescence enveloped by the upper leaves and by the presence of distinctly convex bracts.

#### Type.

Colombia, Putumayo: 15 km S of Villagarzón on road to Puerto Umbria, 360 m, 17 Jul 2018, *Maas*, *Maas-van de Kamer*, *Valderrama*, *Specht*, *Erkens & Rodriguez 10707* (holotype. L, 2 sheets).

#### Description.

***Herb*** 3–5 m tall, young shoots and young leaves glaucous. ***Leaves*** sheaths ca. 20 mm diam; ligule truncate to obliquely truncate, 20–45 mm long; petiole 20–22 mm long, blushed in pink; sheaths, ligule and petiole glabrous; lamina narrowly elliptic, 31– 57 × 12–16 cm, adaxially pale green and glabrous, abaxially green or purple in very young plants, glabrous to sparsely puberulous, base acute to slightly cordate, apex acuminate (acumen ca. 15 mm long). ***Inflorescence*** globose to ovoid, 8–20 × 5–8 cm, wrapped tightly by the upper leaves, terminating a leafy shoot; bracts, bracteole, calyx, ovary, and capsule glabrous. ***Flowers*** adaxially oriented to erect; bracts distinctly convex, reddish on the outer margins, greenish at the centre, coriaceous, ovate, 55–65 × 40–45 mm, apex obtuse, callus green, indistinct; bracteole boat-shaped, 40–45 mm long; calyx red, 15–25 mm long, lobes shallowly triangular to deltate, 4–10 mm long; corolla deep pink, 80–85 mm long, glabrous, lobes 50–60 mm long; labellum yellow, distal edge horizontally spreading, broadly obovate, 60–70 × 60–70 mm, lateral lobes strongly striped with pink or red, middle lobe reflexed, with yellow honey mark, margin irregularly dentate to lobulate; stamen dark pink, 30–40 × 12–15 mm, not exceeding the labellum, apex obtuse, anther 12–15 mm long. ***Capsule*** ellipsoid, 12–30 mm long.

#### Distribution.

Colombia (Putumayo), Ecuador (Morona-Santiago, Zamora-Chinchipe) (Fig. 21H).

#### Habitat and ecology.

In forests, in open shade or sunny areas, often found along roadsides and other disturbed areas, at elevations of 360–1500 m. Flowering year-round.

#### Etymology.

*Costusconvexus* sp. nov. is named for the form of the bracts, which are distinctly convex; thus, the epithet convexus, an adjective meaning “convex or curved outwards” in Latin.

#### Paratypes.

**Colombia. Putumayo**: road to Pto. Guzmán, 300 m, *Skinner R3467.***Ecuador. Morona-Santiago**: near Mendez, 856 m, 17 Oct 2009, Skinner R3196. **Zamora-Chinchipe**: Zamora-Loja road, 1200–1500 m. 3 Feb 1990, *Madsen & Knudsen 86796* (AAU, QCNE); Quebrada del León, affluent of Río Bombuscara, S of Zamora, 1100 m, 5 Feb 1989, Ø*llgaard et al. 90385* (AAU, MO), near entrance to P. N. Podocarpus, 970 m, *Skinner R3331*.

#### Notes.

Plants of *Costusconvexus* sp. nov. can grow to be huge plants, up to 5 m tall, with large inflorescences and flowers. The younger shoots and leaves usually have a glaucous covering making them appear similar to *C.glaucus* Maas but having an inflorescence with distinctly convex bracts that are reddish or pink instead of the pale green and glaucous bracts in *C.glaucus*. It is also characterized by having upper leaves that wrap tightly around and, in doing so, completely hide the young inflorescence.

This new species is observed to be very common along the eastern flanks of the mountains from Putumayo, Colombia, south through Ecuador to Zamora-Chinchipe. Many more recent observations of this new species are recorded on iNaturalist.com in Napo and Pastaza, Ecuador.

### ﻿ Costus douglasdalyi

Taxon classificationPlantaeZingiberalesCostaceae

Maas & H.Maas sp. nov.

C638FD6C-4ABB-5997-B82B-51E018F851AE

urn:lsid:ipni.org:names:77316090-1

#### Diagnosis.

**Figure 11.**
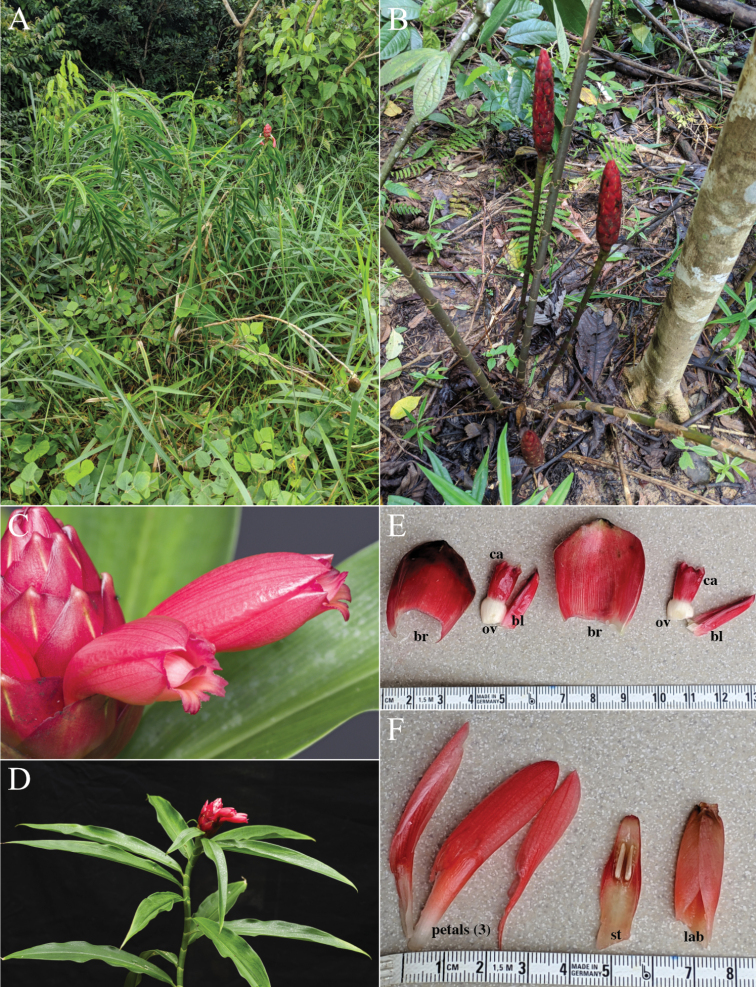
*Costusdouglasdalyi* Maas & H.Maas **A** plant in habitat, inflorescence with flowers on a leafy shoot **B** in habitat, inflorescence on separate leafless shoot **C** flowers **D** plant in cultivation showing narrow leaves **E, F** bract and flower details showing bracts (br), bracteoles (bl), calyx (ca), ovaries (ov), three petals (petals), the fertile stamen (st) and the labellum (lab) **A, B** taken in Cruzeiro do Sul, Acre, Brazil **C–F** photos from plant pressed and accessioned as *D.Skinner R3482* in cultivation. Photos **A–F** by Dave Skinner.

*Costusdouglasdalyi* sp. nov. (Fig. 11) can be confused with *C.erythrothyrsus* Loes., but differs from that species by its very narrow, often almost linear leaves.

#### Type.

Brazil Acre: Mun. Tarauacá, Basin of Rio Juruá, Rio Tarauacá, Seringal Sumaré, 19 Nov 1995, *Daly*, *Silveira*, *Costa*, *Oliveira*, *Lima*, *Figueiredo & Ehringhaus 8606* (holotype U1225319, isotype NY).

#### Description.

***Herb*** 1–4 m tall. ***Leaves*** sheaths 5–20 mm diam; ligule truncate, 2–3 mm long; petiole 3–8 mm long; sheaths, ligule and petiole glabrous to sparsely puberulous; lamina narrowly elliptic to linear, 20–35 × 2–7 cm, abaxially sometimes purple, glabrous to sparsely puberulous, primary vein sparsely covered with a row of erect hairs, adaxially glabrous, base acute to rounded, apex long-acute. ***Inflorescence*** ovoid to cylindric, 8–9 × 4 cm, enlarging to 13–17 × 5–7 cm in fruit, terminating a leafless shoot 45–70 cm long or rarely terminating a leafy shoot, sheaths obliquely truncate, 3–5 cm long, glabrous; bracts, bracteole, calyx, ovary, and capsule glabrous, apex of ovary and fruit sometimes sparsely puberulous. ***Flowers*** abaxially oriented; bracts red to dark red, coriaceous, broadly ovate, 2.5–4 × 2–3 cm, apex obtuse, callus 5–7 mm long; bracteole boat-shaped, 20–25 mm long; calyx red, 9–13 mm long, lobes shallowly triangular, 1–2 mm long; corolla red, pink, or pinkish orange, 45–55 mm long, glabrous, lobes narrowly ovate-elliptic to ovate-elliptic, 35–45 mm long; labellum pink to red, lateral lobes rolled inwards and forming a curved tube ca. 8 mm diam, broadly obovate to elliptic when spread out, 18–40 × 12–30 mm, irregularly 3–7-lobulate, lateral lobes striped with red, middle lobe yellow, 3–5 mm long, irregularly crenulate; stamen pale pink, 15–40 × 7–10 mm, not exceeding the labellum, apex acute, anther 8–10 mm long. ***Capsule*** ellipsoid, 10–15 mm long.

#### Distribution.

Peru (Ucayali), Brazil (Acre, Rondônia) (Fig. 21I).

#### Habitat and ecology.

In non-inundated (terra firme) forests, campinarana, river margins, or roadsides at elevations of 0–350 m. Flowering year-round.

#### Etymology.

This species is named for our dear colleague and friend Douglas Daly, whom PM and HM met many times in his home institute at the New York Botanical Garden Herbarium (NY) and who enabled us to undertake field work in the Brazilian regions of Acre and Amazonas. He also inspired CDS during her graduate studies at the New York Botanical Garden, providing insights into excellence in field research and tropical botany.

#### Paratypes.

**Brazil. Acre**: along road from Cruzeiro do Sul to Barão do Rio Branco, NW of Cruzeiro do Sul, vicinity of São Francisco, 150 m, 25 Aug 1986, *Croat & Rosas Jr. 62674* (MO, U); Mun. Cruzeiro do Sul, vicinity of Cruzeiro do Sul, between airport and downtown, headquarters of Rondon Project, 10 Nov 2001, *Croat 85008* (MO, NY); Mun. Cruzeiro do Sul, km 6 of road from Cruzeiro do Sul to Boa Fé, Ramal dos Carobas, 16 Oct 2001, *Maas et al. 8999* (NY, U); Porangaba, Rio Juruá-Mirim, 15 May 1971, *Maas et al. P 12972* (INPA, NY, U); Porangaba, Rio Juruá-Mirim, 21 May 1971, *Maas et al. P 13213* (INPA, NY, U); Cruzeiro do Sul, Sub-base do Projeto RADAM/BRASIL, próximo ao Aeroporto novo, 23 Feb 1976, *Monteiro & Damião 601* (INPA, MG, U); vicinity of Serra da Moa, 22 Apr 1971, *Prance et al. 12230* (INPA, NY, U). **Rondônia**: Mineração Taboca, proximo ao Campo de Pouso da Mineração 10 Oct 1979, *M.G.G. Vieira et al. 369* (L, NY). **Peru. Ucayali**: Prov. Coronel Portillo, Distr. Caleria, Quebrada Pumayaquillo, left margin of Río Utiquina, 150–175 m, 10 Apr 2003, *Schunke V. & Graham 15567* (U); Prov. and Distr. Padre Abad, carretera al caserio San Miguel y Mapuya, 12–17 km de la Aguaytia, 350 m, 1 Oct 2004, *Schunke V. & Graham 16185* (U).

#### Notes.

*Costusdouglasdalyi* looks quite similar to *C.erythrothyrsus* Loes. in its floral characters but differs by having very narrow, often linear leaves.

### ﻿ Costus gibbosus

Taxon classificationPlantaeZingiberalesCostaceae

D.Skinner & Maas sp. nov.

28BF304A-5167-552F-950A-717D88578DAF

urn:lsid:ipni.org:names:77316091-1

#### Diagnosis.

**Figure 12.**
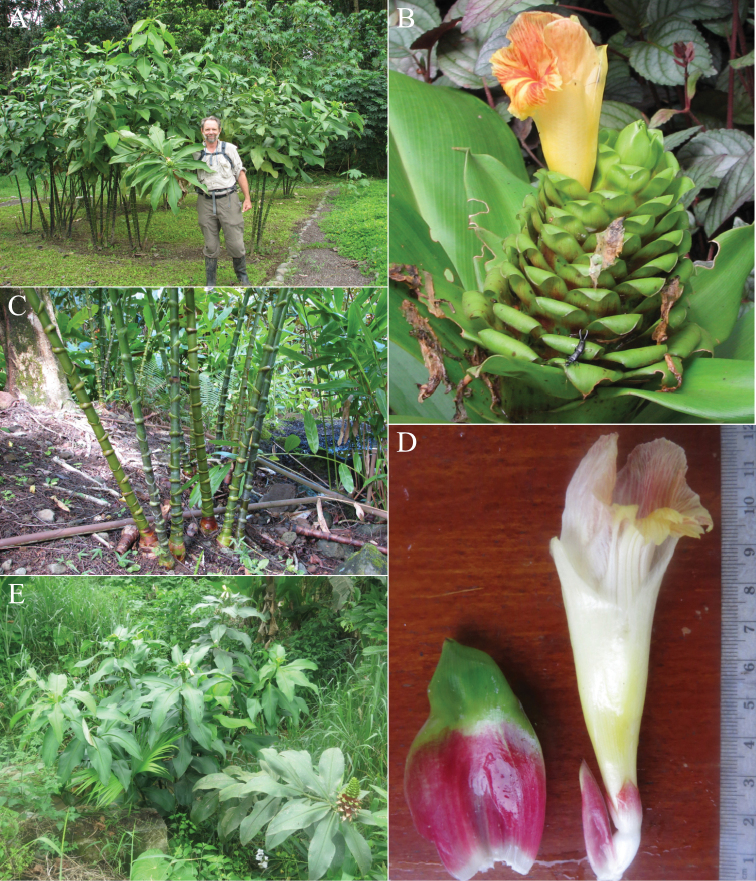
*Costusgibbosus* D.Skinner & Maas **A** plant in cultivation at Rio Palenque Science Center, Ecuador with Dave Skinner **B** inflorescence showing the recurved apices of the bract appendages **C** lower leaf sheaths showing swollen margins **D** close-up of bract (left) and flower (right) **E** plant in habitat near Caluma, El Oro, Ecuador **B, C** at Waimea Arboretum, Hawaii 74S2037, grown from seeds collected by Tim Plowman **D, E** Photos of plant in the wild prior to collecting seeds for *D.Skinner R3346*, currently in cultivation. Photos **A–E** by Dave Skinner.

*Costusgibbosus* sp. nov. (Fig. 12) can readily be recognized by the swollen margin of the lower leaf sheaths; the inflorescence is similar to that of Costusguanaiensis Rusby var. macrostrobilus (K.Schum.) Maas, from which it differs by the horizontal orientation of the bract appendages with often incurved apex and the mostly glabrous leaves, bracts, and calyx.

#### Type.

Ecuador, Pichincha: along the road from Aloag to Santo Domingo, 1150 m (“3500 ft”), 17 Nov 1974, *Plowman & Davis 4455* (holotype U1212018; isotypes GH, USM).

#### Description.

***Herb*** 1.5–5 m tall. ***Leaves*** sheaths 15–35 mm diam, the margin of the lower ones swollen; ligule truncate to slightly 2-lobed, often with a ridge near the apex, 7–14 mm long; petiole 10–15 mm long; sheaths, ligule and petiole glabrous to densely puberulous; lamina narrowly elliptic, 30–50 × 10–20 cm, slightly shiny above, pale green to slightly glaucous below, adaxially glabrous, rarely puberulous, abaxially glabrous, sometimes densely puberulous to villose, base acute to cordate, apex acuminate (acumen 15–20 mm long) to acute. ***Inflorescence*** ovoid to cylindric, 11–18 × 5–9 cm, terminating a leafy shoot; bracts, bracteole, and calyx glabrous, rarely rather densely puberulous, ovary and capsule glabrous to densely puberulo-sericeous. ***Flowers*** abaxially oriented; bracts red, coriaceous, broadly ovate, 40–50 × 30–40 mm; appendages green, foliaceous, erect, broadly triangular to triangular, 15–60 × 10–30 mm, often incurved at the apex; bracteole boat-shaped, 22–32 mm long; calyx red to pink, rarely white, (7–)12–20 mm long, lobes deltate to shallowly triangular, 2–5 mm long; corolla pale yellow, white, or pink, 60–70 mm long, glabrous, lobes ellliptic, 45–60 mm long; labellum pale yellow, orange, pinkish, or white, distal edge horizontally spreading, broadly obovate, 60–85 × 50–70 mm, lateral lobes erect, more or less striped with pink or red, middle lobe reflexed, with yellow honey mark, margin irregularly dentate to lobulate; stamen pink, red, yellow, or white, 40–50 × 12–15 mm, not exceeding the labellum, apex irregularly dentate, anther 9–13 mm long. ***Capsule*** ellipsoid to narrowly ellipsoid, 12–20 mm long.

#### Distribution.

Ecuador (El Oro, Esmeraldas, Guayas, Loja, Manabi, Pichincha) (Fig. 22A).

#### Habitat and ecology.

In forested roadsides, dry forests, or gallery forests at elevations of 0–1500 m. Flowering year-round.

#### Etymology.

*Costusgibbosus* sp. nov. is named for the swollen upper margin of the lower leaf sheaths.

#### Paratypes.

**Ecuador. Bolivar**: Caluma, Pasagua Road, 836 m, *Skinner R3344*, Caluma-El Mirador, 185 m, *Skinner R3346.***El Oro**: between Piñas and Piedras, 1000 m, *Asplund 18160* (S); Road from Santa Rosa to Piñas, 3 km E of Playón, 8 Feb 1987, 350 m, *Bohlin 1226* (GB, QCA); Camino a Limón-Playa, cerca a Río Dumari, 600 m, 12 Oct 1993, *Cornejo & Bonifaz 425* (AAU, QCNE); Road Zaruma-Santa Rosa, between Piñas and El Placer, 800 m, 6 May 1974, *Harling & Andersson 14354* (GB, U). **Esmeraldas**: Hacienda Timbre, 28 May 1955, *Asplund 16494* (S); along road to Río Tulubí from main San Lorenzo-Lita Highway, 12 July 2000, 59 m, *Croat 83948A* (MO, QCNE); between Esmeraldas and Tabiazo, 0 m, 14 Sep 1977, *Maas et al. 2915* (QCA, U). **Guayas**: Guayaquil, Cerro Azul, *Asplund 15412* (S); Cerro Azul, W of Guayaquil, 25 Mar 1955, *Asplund 15895* (NY, S); Cordillera Chongón-Colonche, Río California, 150 m, 27 Sep 1997, *Cornejo & Bonifaz 5792* (GUAY, U); Cerro Azul Cordillera Chongon-Colonche, 12 km W of Guayaquil, Quebrada Canoa, 17 Jan 1991, 50–300 m, *Gentry & Josse 72332* (F, MO, QCNE); Teresita, 3 km W of Bucay, 270 m, 5 Jul 1923, *Hitchcock 20543* (GH, NY, US); Bosque Protector Cerro Blanco, Carretera Guayaquil-Salinas, km 17, 200–440 m, 31 Oct 1995, *T. Nuñez 306* (MO, QCNE); Cantón Guayaquil, Bosque Protector Cerro Blanco, carretera a Salinas, km 15, 100 m, 19 Aug 1991, *Rubio et al. 2007* (MO, QCNE, U). **Loja**: Macará-Cariamanga Road, ca. 3 km NE of Sabiango, 900–1000 m, 17 Apr 1980, *Harling & Andersson 18358* (S, U). **Los Ríos**: Río Palenque Biological Station, 220 m, 17 Sep 1973, *Dodson & Tan 5347* (F, QCA, US); Río Palenque Science Center, km 56 of Quevedo-Santo Domingo Road, 14 May 1982, *Dodson 13039* (MO, SEL); Río Palenque Field Station, halfway between Quevedo and Santo Domingo de los Colorados, 200 m, 24 Feb 1974, *Gentry 10165* (MO, RPSC, SEL). **Manabi**: 40 km along road from Santo Domingo de los Colorados to Chone, 19 May 1955, *Asplund 16421* (S); El Aromo, 13 km SW to San Mateo, then E 13 km to town, 100 m, 4 Dec 1986, *Hammel & Trainer 15871* (MO); road Chone-El Carmén, at Flavio Alfaro, 350 m, 28 Oct 1980, *Holm-Nielsen et al. 27916* (AAU). **Pichincha**: shore of Río Pilatón, at the bridge of the road from Chiriboga to Santo Domingo de los Colorados, 1000 m, 2 Jul 1955, *Asplund 16763* (S); Santo Domingo de los Colorados, 530 m, 19 Jun 1975, *Gilli 119* (W); new Alluriquín-Quito Road, km 6, 850 m, 10 Oct 1980, *Maas & Cobb 4795* (NY, QCA, U); Santo Domingo de los Colorados, 200 ft, 28 Oct 1960, *Pennington 56 SD* (K, NY); along the road from Aloag to Santo Domingo, Tandape, 1150 m (“5000 ft”), 17 Nov 1974, *Plowman & Davis 4454* (GH, U); Toachi, on road Aloag-Santo Domingo, 750 m, 3 Jan 1967, *Sparre 13862* (S, U); Carretera Quito-Puerto Quito, km 113, near Reserva de ENDESA “Corporación Forestal Juan Manuel Durini”, 800 m, 4 Mar 1984, *Ulloa U. 111* (QCA, U).

#### Notes.

*Costusgibbosus* sp. nov. is easily recognized in the field by the swollen margins of the lower leaf sheaths.

### ﻿ Costus mollissimus

Taxon classificationPlantaeZingiberalesCostaceae

Maas & H.Maas sp. nov.

39BB13CA-64F0-54F7-B933-299D0FB382DB

urn:lsid:ipni.org:names:77316092-1

#### Diagnosis.

**Figure 13.**
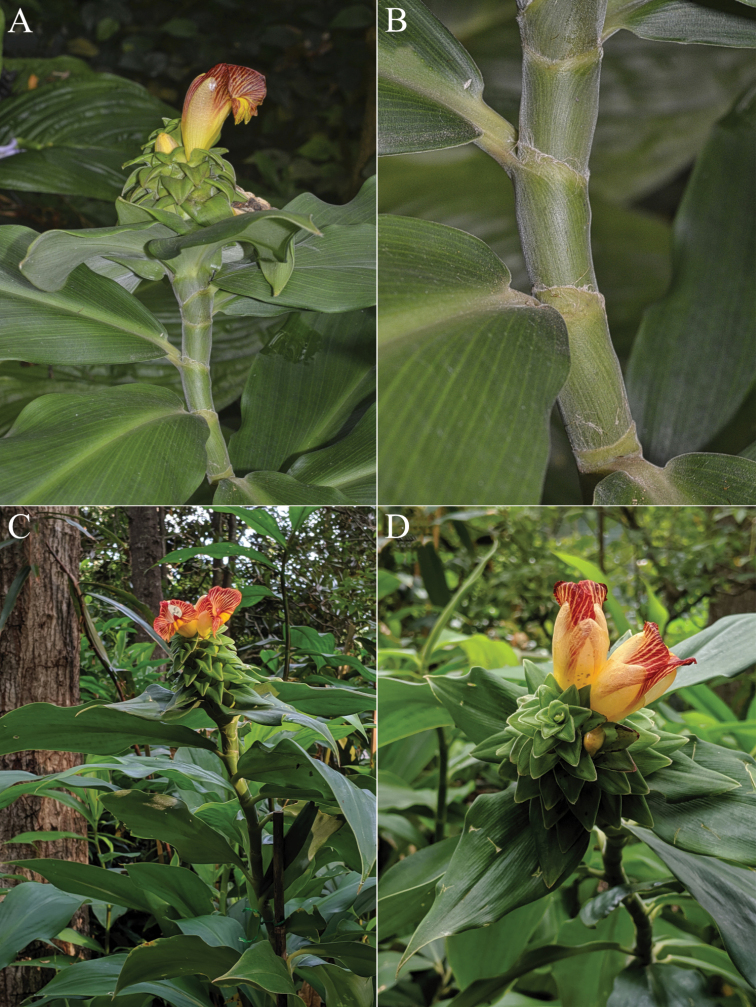
*Costusmollissimus* Maas & H.Maas **A** inflorescence **B** ligules and sheath, showing short ligule and indument **C, D** cultivated plant growing in Florida, propagated from living accession *1998GR01567* at Utrecht Botanical Garden. Photos **A, B** by Paul Maas **C, D** by Dave Skinner.

*Costusmollissimus* sp. nov. (Fig. 13) shares with *C.dirzoi* Garcia-Mend. & G.Ibarra a distinct velutinous indument on most parts of the plant, but differs by having appendaged bracts and a very short ligule. Moreover, *C.dirzoi* has bracts with a distinct callus, absent in *C.mollissimus*.

#### Type.

Mexico, Chiapas: Mun. Chilon, 22 km from Temo in the direction of Palenque, 2 km from Patathel, near El Chich, 850 m, 4 Nov 1998, *Ishiki Ishihara*, *Maas & Maas-van de Kamer 2400* (holotype L, 2 sheets: L4368349 & L436835; isotype ECOSUR).

#### Description.

***Herb*** 0.5–2.5 m tall. ***Leaves*** sheaths 12–15 mm diam; ligule truncate, 1–4 mm long; petiole 10–15 mm long; sheaths, ligule and petiole densely velutinous; lamina narrowly elliptic, sometimes elliptic, 18–36 × 6–13 cm, slightly 5–8-plicate, adaxially shiny, glabrous, abaxially densely velutinous, base acute, obtuse, to cordate, apex acuminate (acumen 5–15 mm long). ***Inflorescence*** cylindric to subglobose, 5–13.5 × 4–5 cm, terminating a leafy shoot; bracts, appendages of bracts, bracteole, calyx, ovary, and capsule densely velutinous. ***Flowers*** abaxially oriented; bracts green, red, or pink, coriaceous, broadly elliptic, 2–2.5 × 2–3 cm; appendages green, foliaceous, patent to slightly reflexed, triangular to broadly triangular, 0.5–3.5 × 1.2–2.5 cm, apex acute to obtuse; bracteole boat-shaped, 16–20 mm long; calyx red to pink, 9–10 mm long, lobes broadly to shallowly triangular, 2–5 mm long, with a hairy tuft at the apex; corolla cream, yellow, or pale orange, 50–60 mm long, densely velutinous, lobes narrowly elliptic, 30–40 mm long; labellum yellow, distal edge horizontally spreading, broadly obovate, 30–40 × 40–50 mm, lateral lobes dark red with yellow venation, middle lobe reflexed with yellow honey mark, margin dentate; stamen yellow, 30–35 × 10–12 mm, not exceeding the labellum, apex red, slightly 3-dentate, anther ca. 8 mm long. ***Capsule*** broadly obovoid to obovoid, 12–17 mm long.

#### Distribution.

Mexico (Chiapas, Oaxaca, Tabasco, Veracruz) (Fig. 22B).

#### Habitat and ecology.

In forests (a.o. “selva baja/alta perennifolia”) with *Brosimum* sp., *Bursera* sp., *Calophyllum* sp., *Cymbopetalum* sp., *Dialium* sp., *Ficus* sp., *Tapirira* sp., and *Sloaneatuerckheimii* Donn.Sm., at elevations of 200–1000(–1500) m. Flowering year-round.

#### Vernacular name.

Mexico: Apagafuego.

#### Etymology.

This species is named after its very soft indument on the abaxial surface of the lamina, the word “mollis” meaning soft in Latin.

#### Paratypes.

**Mexico. Chiapas**: Mun. Solosuchiapa, 3–5 km above Solosuchiapa, along road to Tapilula, 450 m, 26 Jul 1972, *Breedlove 26463* (MO); Mun. Ixcomitan, 2 km E of Ixcomitan, road from Villahermosa to Tuxtla Gutierrez, 550 m, 11 Feb 1983, *Martínez Salas et al. 3160* (MEXU); Mun. Pichucalco, 10–12 km S of Pichucalco, 8 Mar 1983, *Ramamoorthy et al. 1790* (MEXU); NW end of Valley of Chiapas on road to Mal Paso, 41 km (by road) NW of Ocozocoautla, 350 m, 4–5 Aug 1965, *Roe et al. 928* (WIS). **Oaxaca**: Mun. Santa María Chimalapa, Arroyo Milagrito, ca. 6 km SE of Santa Maria, 1 km from Vereda, which passes Arroyo Sanate, 230 m, 30 Aug 1984, *Hernández G. 387* (MEXU, MO); Mun. Santa María Chimalapa, ca. 15 km ESE of Santa María, Vereda near Arroyo Plata, loma S of Río Milagro, 400 m, 3 Sep 1985, *Hernández G. 1435* (MEXU, MO). **Tabasco**: near San Manuel, S of village along Río Mezcalapa, 11 Sep 1944, *Gilli & Hernandez Xolocotzi 97* (MEXU). **Veracruz**: Las Cruces, Las Choapas, 250 m, 14 Jul 1970, *Nevling & Gomez-Pompa 1526* (F).

#### Notes.

*Costusmollissimus* sp. nov. superficially resembles *C.dirzoi* García-Mend. & G.Ibarra, as both share the velutinous indument all over the plant, but differs in having bracts with foliaceous, green appendages and a shorter ligule (1–4 mm vs. 3–12 mm long). *Costusdirzoi* has bracts with a distinct callus that is absent in *C.mollissimus*.

In Maas’ treatment of Costaceae (“Costoideae”) for Flora Neotropica (Maas 1972: 57), this species was erroneously included in C.guanaiensis Rusby as var. tarmicus (Loes.) Maas.

### ﻿ Costus obscurus

Taxon classificationPlantaeZingiberalesCostaceae

D.Skinner & Maas sp. nov.

21E5187F-3300-5474-9433-14D0CC978B38

urn:lsid:ipni.org:names:77316093-1

#### Diagnosis.

**Figure 14.**
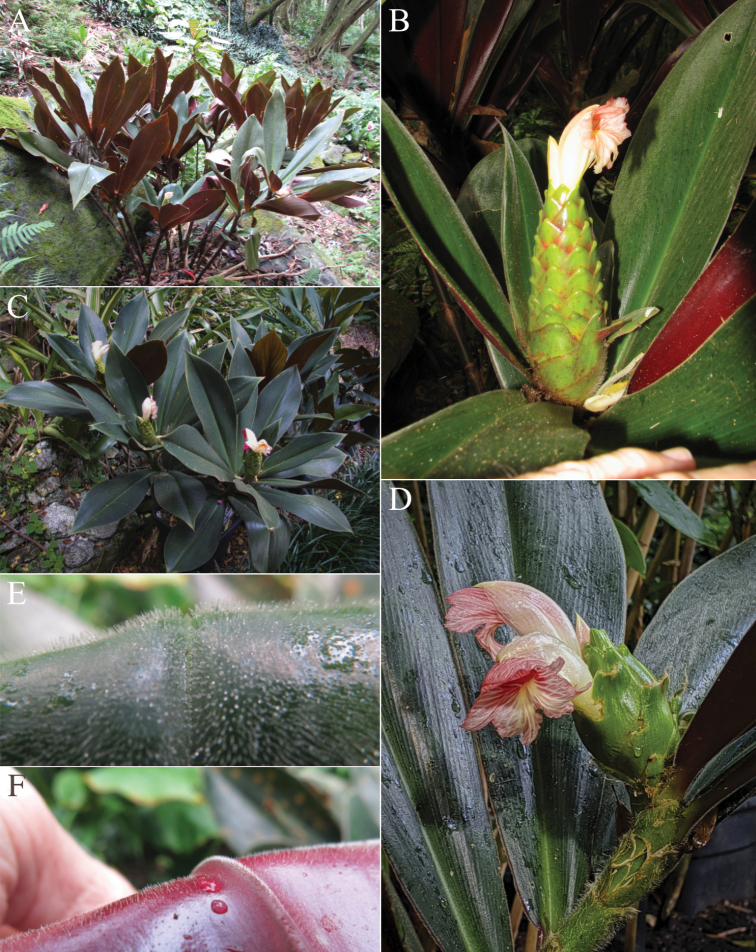
*Costusobscurus* D.Skinner & Maas **A** cultivated plant from Plowman’s type collection showing upright orientation of leaf lamina **B** inflorescence **C** cultivated plant showing colours and upright orientation of leaf lamina **D** photo showing villose indument on leaf sheaths **E** Indument on adaxial leaf surface **F** indument on abaxial leaf surface **A, B** cultivated specimen from Lyon Arboretum, Hawaii *L-81.0901* grown from seeds wild collected by Tim Plowman from the plant accessioned as *Plowman 5054***C–F** photos taken in Pantiacolla, Madre de Dios, Perú prior to collecting seeds for *D.Skinner R3322*, currently in cultivation. Photos **A–F** by Dave Skinner.

*Costusobscurus* sp. nov. (Fig. 14) can be recognized by its large, dark green leaves with dark purple undersides and densely whitish villose sheaths. It could superficially be confused with *C.erythrophyllus* Loes. and *C.acreanus* (Loes.) Maas, but these species never have densely villose sheaths.

#### Type.

Peru, Madre de Dios: Prov. Manu, near Shintuya, on road to Salvación, 600 m, 9 Feb 1974, *Plowman & Davis 5054* (holotype USM; isotypes GH, L1483069, L1480371, L1480372).

#### Description.

***Herb*** 1–1.5 m tall. ***Leaves*** sheaths 10–20 mm diam; ligule truncate, 10–15 mm long; petiole 8–12 mm long; sheaths, ligule and petiole densely to rather densely whitish villose; lamina narrowly elliptic, 32–45 × 9–17 cm, adaxially dark green, rather densely to densely whitish villose, abaxially dark purple, densely to sparsely whitish puberulous, base acute to cordate, apex acuminate (acumen 5–10 mm long). ***Inflorescence*** ovoid, 8–13 × 5–7 cm, terminating a leafy shoot; bracts, appendages of bracts, bracteole, calyx, ovary, and capsule rather densely to sparsely puberulous. ***Flowers*** abaxially oriented; bracts green in the exposed part, red or green in the covered part, coriaceous, broadly ovate-triangular, 35–45 × 30–35 mm; appendages green, erect, broadly ovate-triangular, 15–20 × 18–20 mm, or almost absent, apex rounded; bracteole boat-shaped, 35–37 mm long; calyx red, 25–27 mm long, deeply split on one side, lobes deltate to broadly triangular, ca. 2 mm long; corolla cream to yellow, ca. 75 mm long, glabrous, lobes narrowly elliptic, 55–60 mm long; labellum pale yellow, distal edge horizontally spreading, broadly obovate, ca. 60–70 × 60–70 mm, lateral lobes strongly striped with pink or red, middle lobe reflexed, with yellow honey mark, margin irregularly dentate to lobulate; stamen white to pink, 35–45 × 12–15 mm, not exceeding the labellum, apex pinkish red, deeply and irregularly lobed, anther 7–8 mm long. ***Capsule*** ellipsoid, 12–15 mm long.

#### Distribution.

Peru (Cuzco, Huánuco, Madre de Dios, San Martín) (Fig. 22C).

#### Habitat and ecology.

In forests, in wet, shady areas, often in disturbed places, at elevations of 450–1300 m. Flowering in the rainy season.

#### Etymology.

*Costusobscurus* sp. nov. is known for its leaves having a very dark green adaxial surface and dark purple abaxial surface, and for growing in dark and shady places, hence the specific epithet obscurus (‘obscurus’ means ‘dark, shady, “indistinct’ in Latin). This species might also be considered “obscure” (in English: unclear, uncertain, unknown, or in doubt) because it has been confused by the authors in the past as either *C.erythrophyllus* Loes. or as *C.acreanus* (Loes.) Maas. Only recently has it been determined to be an undescribed species.

#### Paratypes.

**Peru. Cuzco**: Prov. La Convención, Distr. Echarati, Cashiriari-3 well site, 5 km S of Camisea River, 700 m, 2 Sep 1998, *Nuñez V. et al. 23785* (F). **Huánuco**: Prov. Leoncio Prado, Puente Pucayaco, Río Pucayaco, road from Tingo Maria to Tocache, 570 m, 30 Mar 1976, *Plowman & Kennedy 5775* (U); cultivated in Tocache, besides Maior Plaza, originally collected by José Schunke V. at Quebrada Cachiyaco de Lopuna, near Mina de Sal, S of Tocache, 31 Mar 1976, *Plowman & Kennedy 5791* (GH, U); cultivated in Jardin Botanico de Tingo Maria, from material collected by Plowman in Ramal de Aspusana, 65 km N of Tingo Maria, on the border of Huánuco and San Martín, 5 Jul 1978, *Plowman & Ramírez R. 7595* (F, U). **Madre de Dios**: Prov. Manu, Cerro de Pantiacolla, Río Palotoa, 10–15 km NNW of Shintuya, 700–1300 m, 16 Dec 1985, *Foster et al. 10937* (F); Pantiacolla, serrania across Río Alto Madre de Dios from Shintuya, 450–650 m, 28 Oct 1979, *Gentry 27304* (MO, U). **Cultivated material**: Harvard Forest Greenhouses, Petersham, Massachusetts, USA, cultivated from *Plowman & Davis 5054*, Peru, *Plowman & Davis 5054 A* (F, L); cultivated from Peru, Huánuco, 8 km NW of Tingo Maria, along quebrada on road to Shinchi Roca, 900 m, 14 Nov 2016, *Skinner R3383* (BH). Peru, Madre de Dios, Pantiacolla, 25 Jan 2013, *Skinner R3322* (UC).

#### Notes.

Plants of *Costusobscurus* sp. nov. have a compact appearance, with leaves closely spaced together along the shoot and generally pointed upward rather than horizontally or drooping. The leaves’ dark green adaxial surface and dark purple abaxial surface are striking in appearance. *Costusobscurus* can be confused with *C.erythrophyllus* Loes., but can be distinguished from that species by its shorter, truncate ligule instead of a long and deeply 2-lobed ligule as well as by having smooth rather than (mostly) plicate leaves.

This new species has been widely cultivated from the type collection *Plowman 5054*. Plowman’s journal indicates the distribution of live plants to Marie Selby Botanical Garden and Lyon Arboretum, which is accessioned as L-81.0901. It has sometimes been confused with the famous “El Whiskey” plant from Colombia, but it can be easily distinguished by the indumenta on the adaxial and abaxial leaf surfaces.

### ﻿ Costus oreophilus

Taxon classificationPlantaeZingiberalesCostaceae

Maas & D.Skinner sp. nov.

69D7C492-E441-55FE-9E3C-CCCA0798E02C

urn:lsid:ipni.org:names:77316094-1

#### Diagnosis.

**Figure 15.**
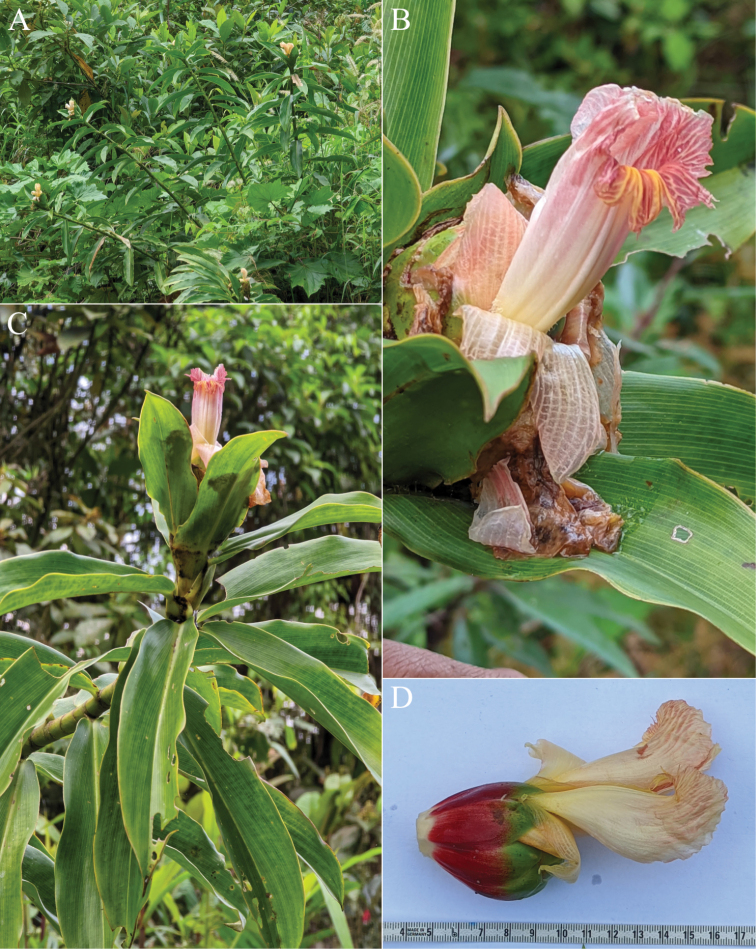
*Costusoreophilus* Maas & D.Skinner **A** plant in habitat **B** inflorescence showing clasping upper leaves and recurved corolla lobes on a fresh flower **C** flowering shoot showing narrowly elliptic leaf lamina **D** upper part of inflorescence showing recurved corolla lobes and adaxially oriented to erect labellum. Photos **A–D** by Dave Skinner taken near La Molienda along the Río Estancia, Tungurahua, Ecuador.

*Costusoreophilus* sp. nov. (Fig. 15) can be confused with *C.laevis* Ruiz & Pav., with which it shares the mostly green bracts and adaxially oriented flowers, but it is distinguished by the puberulous sheaths and leaves and a smaller ligule (3–10 mm vs. 5–20 mm long); moreover the corolla lobes are often recurved, a feature rarely seen in *Costus*.

#### Type.

Ecuador, Tunguruhua: Río Verde Grande, 1500 m, 30 Mar 1956, *Asplund 20068* (holotype S, 2 sheets S06-1367 & S06-13643; isotype GB).

#### Description.

***Herb*** 3–4 m tall. ***Leaves*** sheaths 15–20 mm diam; ligule truncate, 3–10 mm long; petiole 5–10 mm long; sheaths, ligule and petiole densely to sparsely puberulous; lamina narrowly elliptic, 20–38 × 7–11 cm, adaxially shiny, glabrous, sometimes rather densely puberulous, abaxially green, densely to sparely puberulous to velutinous, base cordate to acute, apex acuminate (acumen 5–10 mm long). ***Inflorescence*** cylindric to ovoid, 7–12 × 3–6 cm, wrapped tightly by the upper leaves, terminating a leafy shoot; bracts, bracteole, calyx, ovary, and capsule sparsely puberulous to glabrous. ***Flowers*** adaxially oriented to erect; bracts green in the exposed part, red in the covered part, sometimes completely red, coriaceous, broadly ovate, 3.5–4.5 × 3–4 cm, apex obtuse, callus inconspicuous; bracteole boat-shaped, 18–31 mm long; calyx red, 6–12 mm long, lobes shallowly triangular, 3–4 mm long; corolla white to yellow, 50–60 mm long, glabrous, lobes recurved, narrowly obovate, 40–55 mm long; labellum white, distal edge horizontally spreading, broadly obovate, 70–75 × 70–75 mm, lateral lobes striped with red, middle lobe reflexed, with yellow honey mark, margin irregularly crenulate; stamen red to pink, 35–40 × 12–13 mm, not exceeding the labellum, apex red, irregularly dentate, anther 10–12 mm long. ***Capsule*** ellipsoid, c. 15 mm long.

#### Distribution.

Ecuador (Pastaza, Tungurahua, Zamora-Chinchipe) (Fig. 22D).

#### Habitat and ecology.

In forests, at elevations of 1150–1600 m. Flowering in the rainy season.

#### Etymology.

The species name “*oreophilus*” is derived from the Greek words *oros* (= mountain) and *philos* (= beloved) as this is a mountain-loving species.

#### Paratypes.

**Ecuador. Pastaza**: road to Puyo, km 45–60, 1300–1400 m, 8 Oct 1961, *Dodson & Thien 913* (WIS). **Tungurahua**: valley of Río Pastaza, Hacienda Verde Grande, 1500 m, 26 Jul 1939, *Asplund 7833* (GB, S); between Río Mapoto and Río Margaritas, along Canelos trail, 1225 m, 20 Mar 1939, *Penland & Summers 179* (BM, F, MO). **Zamora-Chinchipe**: along road Loja-Zamora, km 45–51, 1400–1600 m, 20 Nov 1961, *Dodson & Thien 1441* (WIS). **Cultivated material**: Ecuador, Zamora Chinchipe, Parque Nacional Podocarpus, 1175 m, 25 Jan 2013, *Skinner R 3332*; Ecuador, Zamora Chinchipe, Río Numbamia, 1270 m, *Skinner R 3333*.

#### Notes.

Most collections of *Costusoreophilus* sp. nov. were placed by Maas (1972) under *Costuslaevis* Ruiz & Pav. with a note saying: “Three Ecuadorian collections deviated by being densely puberulous to velutinous on the lower side [abaxial surface] of the leaves. The flower material being incomplete, I could not determine their taxonomic status, i.e., whether it is a variety of *C.laevis* or a distinct species”. Now, 50 years later, we recognize these specimens merit specific rank. *Costusoreophilus* differs from *C.convexus* sp. nov. Maas & Skinner by various features such as the hairy instead of glabrous sheaths and leaves, a smaller calyx (6–12 vs. 15–25 mm long), a smaller ligule (3–10 vs. 20–45 mm long), and recurved corolla lobes, a feature quite rare in *Costus*.

The authors are indebted to orchid specialist Marco Jiménez Villata of Zamora, Ecuador, who has explored the forests of Zamora-Chinchipe for over 30 years. He showed Dave Skinner several localities in the province, where he was able to make photographs of this species. Skinner later found the living plants of this new species in Tungurahua, which were consistent with the plants in Zamora-Chinchipe but usually with less dense hairs on the undersides of the leaves. Moreover, in living material, the corolla lobes were found to be sharply recurved, which is very unusual in the genus *Costus*.

### ﻿ Costus pitalito

Taxon classificationPlantaeZingiberalesCostaceae

C.D.Specht & H.Maas sp. nov.

C7973658-9BB9-5B08-846B-F3556F31AB88

urn:lsid:ipni.org:names:77316095-1

#### Diagnosis.

**Figure 16.**
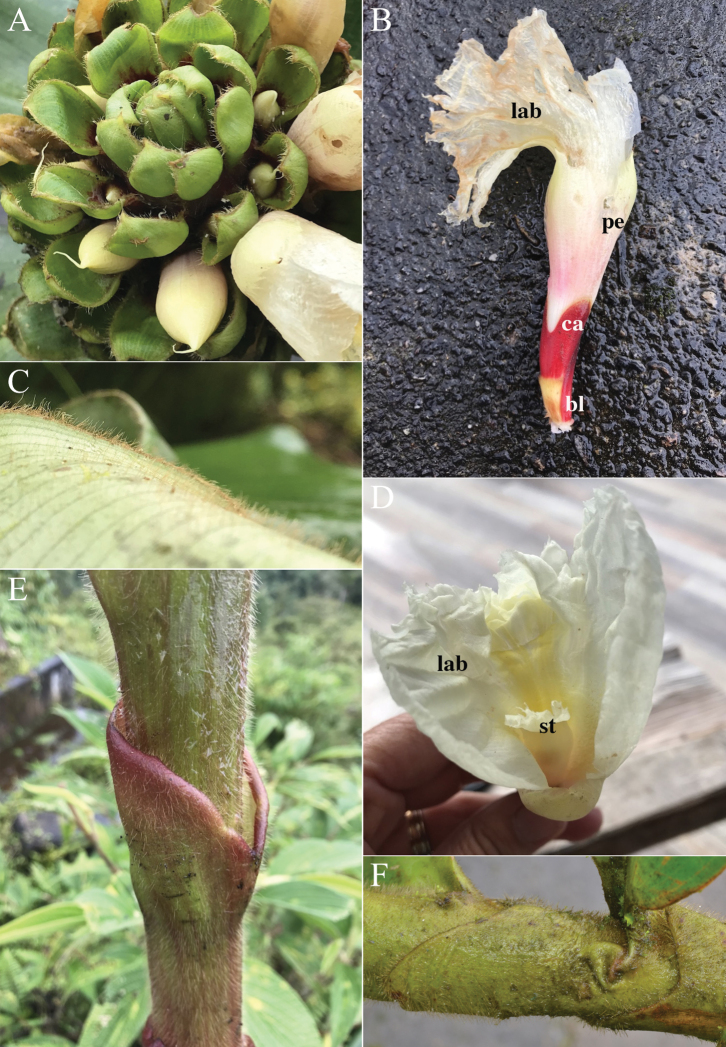
*Costuspitalito* C.D.Specht & H.Maas **A** inflorescence as seen from above showing hairs on the margins of the bracts **B** close-up of single flower with attached bracteole (bl) indicating the calyx (ca), petals (pe) and labellum (lab) **C** indument of abaxial leaf surface **D** flower as viewed from the top showing the open labellum (lab) and single fertile stamen (st) **E** close-up of swollen leaf sheaths **F** leaf ligule. Photos of type specimen *Maas et al. 10733* taken prior to pressing **A–E** by Chelsea Specht, **F** by Paul Maas.

*Costuspitalito* sp. nov. (Fig. 16) looks superficially like *C.leucanthus* Maas based on the shared light-coloured flowers and a very long ligule, but it differs from *C.leucanthus* by markedly obtuse ligule lobes and bracts with densely brownish villose margins.

#### Type.

Colombia, Cauca: forested roadside from San Juan de Villalobos to Pitalito, 1590 m, 19 Jul 2018, *Maas*, *Maas-van de Kamer*, *Valderrama*, *Specht*, *Erkens & Rodriguez 10733* (holotype L, 2 sheets).

#### Description.

***Herb*** 1.7– 2.5 m tall. ***Leaves*** sheaths ca. 35 mm diam; ligule deeply 2-lobed, 45–70 mm long, lobes rounded, obtuse; petiole 10–20 mm long; sheaths, ligule and petiole densely, brownish villose; lamina narrowly elliptic, 30–45 × 12.5–14 cm, adaxially glabrous but sparsely brownish villose when young, abaxially densely brownish villose, base acute, apex acuminate (acumen ca. 10 mm long). ***Inflorescence*** suglobose, 11–12 × 9–11 cm, terminating a leafy shoot; bracts rather densely villose, appendages of bracts sparsely villose to glabrous, its margins densely brownish villose, bracteole and calyx sparsely puberulous, ovary and capsule rather densely sericeous. ***Flowers*** abaxially oriented to erect; bracts red, coriaceous, broadly ovate, 3–4 × 2–3 cm; appendages green, foliaceous, cup-shaped, erect to patent, broadly ovate-triangular, 1–1.5 × 1–1.5 cm, apex obtuse to acute; bracteole 1- to 2-keeled, 30–32 mm long; calyx red, apex green, 20–22 mm long, lobes ovate-triangular, 10–12 mm long; corolla pale yellow to hyaline, base pinkish, 55–60 mm long, glabrous, lobes narrowly elliptic, 35–40 mm long, with filiform apex; labellum light yellow to cream, darker yellow in throat, distal edge horizontally spreading, broadly obovate, ca. 70 × 70–80 mm, margin irregularly dentate to crenate; stamen cream, ca. 50 × 15 mm, apex reflexed, irregularly dentate, not exceeding the labellum, anther ca. 13 mm long. ***Capsule*** ellipsoid, ca. 25 mm long.

#### Distribution.

Colombia (Cauca, Putumayo) (Fig. 22E).

#### Habitat and ecology.

Along forested roadsides, at an elevation of ca. 1600 m. Flowering and fruiting: May and July.

#### Etymology.

This species is named “pitalito” after its type locality near Pitalito, in the Colombian department of Cauca.

#### Paratypes.

**Colombia. Putumayo**: Mun. Villagarzón, carretera a Puerto Asis, 4 May 1994, *Fernández-Alonso et al. 11440* (COL).

#### Notes.

*Costuspitalito* looks superficially like *C.leucanthus* Maas by its white flowers and a very long ligule, but it differs by obtuse ligule lobes and bracts with densely brownish villose margins.

### ﻿ Costus prancei

Taxon classificationPlantaeZingiberalesCostaceae

Maas & H.Maas sp. nov.

28B17AA1-3A49-5833-A724-19FB53764B67

urn:lsid:ipni.org:names:77316096-1

#### Diagnosis.

**Figure 17.**
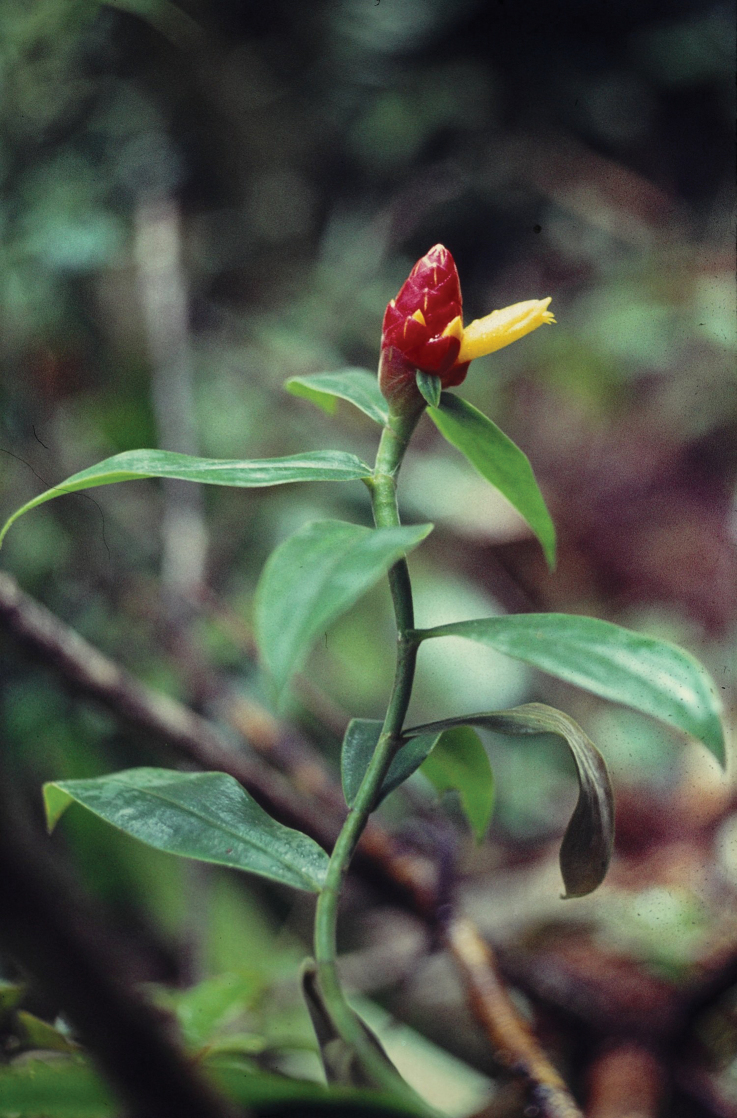
*Costusprancei* Maas & H.Maas Photo in habitat taken by Paul Maas at Serra do Moa, Acre, Brazil. Photo corresponds to *Prance 12265* collected by G.T. Prance in 1971.

*Costusprancei* sp. nov. (Fig. 17) looks quite similar to *C.sprucei* Maas (a species restricted to the Brazilian state of Pará) and has been confused with it in the past, both sharing most of the vegetative and floral characters, but this species has yellow to orange flowers whereas they are pinkish red in *C.sprucei*.

#### Type.

Brazil, Acre: vicinity of Serra da Moa, 22 Apr 1971, *Prance*, *Maas*, *Kubitzki*, *Steward*, *Ramos*, *Pinheiro & Lima 12265* (holotype U, 2 sheets: U1607120 & U1603130 (spirit collection); isotypes INPA, NY00867011).

#### Description.

***Herb*** 1–1.5 m tall. ***Leaves*** sheaths 8–10 mm diam; ligule truncate, 2–7 mm long; petiole 2–8 mm long; sheaths, ligule, and petiole densely to sparsely villose; lamina narrowly elliptic, 11–25 × 4–7 cm, adaxially densely villose to glabrous with a dense row of hairs along the primary vein, abaxially densely puberulous to villose, base acute, rounded, or cordate, apex acuminate (acumen ca. 15 mm long). ***Inflorescence*** cylindrical, 3–15 × 1.5–4 cm, terminating a leafy shoot; bracts, bracteole, calyx, ovary, and fruit densely puberulo-villose to glabrous. ***Flowers*** abaxially oriented; bracts red, coriaceous to chartaceous, broadly to depressed ovate, 1.5–2.5 × 1.5–3 cm, callus 3–5 mm long; bracteole boat-shaped, 9–15 mm long; calyx red to pale orange-red, 6–12 mm long, lobes shallowly triangular, 1– mm long; corolla yellow to orange (2 labels indicated cream and pink to orange!), 20–30 mm long, glabrous, lobes narrowly elliptic, 20–25 mm long; labellum yellow to orange, lateral lobes involute and forming a straight tube 7–8 mm diam, oblong-obovate when spread out, 20–25 × 12–16 mm, irregularly lobulated, lobules 0.5–5 mm long; stamen yellow, 23–25 × 7–8 mm, slightly exceeding the labellum, apex rounded, anther ca. 6 mm long. Capsule ellipsoid to subglobose, 6–7 × 5–7 mm.

#### Distribution.

Brazil (Acre, Amazonas, Rondônia), Peru (Loreto, San Martín) (Fig. 22F).

#### Habitat and ecology.

In non-inundated (terra firme) or periodically inundated (várzea) forests at elevations of 0–260 m. Flowering year-round.

#### Vernacular names.

Brazil: Canafiche.

#### Etymology.

This species is named after our friend and dearest colleague Sir Ghillean Prance, who invited us (PM and HM) in 1971 to join various of his expeditions into the Brazilian Amazon region and who inspired me (PM) very much to continue working as a Neotropical taxonomist.

#### Paratypes.

**Brazil. Acre**: Mun. Mançio Lima, Upper Rio Moa, top of Serra Azul, 12 Oct 1968, *Campbell 8951* (NY, RB); Mun. Mançio Lima, Serra do Moa, 29 Dec 1998. *Ehrich 5c* (L, NY); Mun. Mançio Lima, PARNA, Serra do Divisor, trilha para a Cachoeira Formosa, 225 m, 21 Aug 2008, *Fiaschi 3287* (NY). **Amazonas**: Rio Nhamundá, *Assumpção & Coêlho 72* (INPA); Tapurucuara, Rio Negro, 6 Feb 1959, *Cavalcante 540* (MG); Mun. Humaitá, BR 230, Rodovia Transamazonica, 126 km from Humaitá, 12 Apr 1985, *Cid et al 5437* (INPA, NY); Mun. Manaus, c. 80 km NNE of Manaus, Fazenda Esteio, 50–125 m, 23 Jun 1992, *Nee 42842* (NY, U); Tapurucuara, road to the airport, 16 Oct 1971, *Prance et al. 15300* (COL, DAV, F, INPA, NY, U, US, VEN, W); Rio Canumã, 1 Mar 1945, *Proctor Cooper s.n.* (COL, US); Manaus-Itacoatiara Road, Rio Preto, km 80, 14 Nov 1966, *Prance et al. 3151* (F, MO, NY, U, US); Manaus-Itacoatiara Road, Rio Preto, km 90, 19 Jul 1961, *Rodrigues & Lima 2996* (INPA, U); Tapurucuara, 22 Jan 1978, *Steward et al. 476* (INPA, MO, NY, U, US); Lake Canumã, *Von Martius s.n.* (M). **Rondônia**: Estrada Porto Velho-Cuiabá, km 48, 5 Feb 1983, *Bilby et al. 33* (INPA); SE bank of Rio Jaci Paraná, before Jaci Paraná town, 9 Jul 1979, *C.E. Calderón et al. 2986* (NY, US); Porto Velho, Guajará-Mirim, Estrada do Palheta, km 12, foot of Serra Parecis, 28 Jan 1983, *Carreira 315* (INPA, MG); Itapuã de Oeste, Floresta Nacional de Jamari, 4 Dec 2011, *Castro et al. 81* (herbarium unknown); 1 km from BR 364, ca. 1 km N of road to São Sebastião, 24 May 1984, *Frame 171* (NY, U); Ji-Paraná, Reserva Biológica do Jaru, 125 m, 8 Jun 2015, *Labiak 6250* (NY); between Jaci Paraná and Rio Madeira, 26 Jun 1968, *Prance et al. 5218* (INPA, NY, U, US); basin of Rio Madeira, foothill of Serra dos Pacaás-Novos, 12 km NNE of Guajará-Mirim, 1 Aug 1968, *Prance et al. 6672* (COL, INPA, NY, U); 8 km N of Porto Velho, basin of Rio Madeira, 7 Nov 1968, *Prance et al. 8227* (F, INPA, NY, U); road to São Lorenço mines, N bank of Rio Madeira, 10 km above Mutumparaná, 25 Nov 1968, *Prance et al. 8866* (NY, U, US); Porto Velho, Parque Nacional Mapinguari, trilha do parquet, 12–14 Dec 2013, *Silveira 559*, *596* (L, NY). **Peru. Loreto**: Prov. Requena, Oyo de Contaya, Serra del Divisor, between Contamana and Río Tapiche, 260 m, 10 Aug 2005, *Uliana et al. 1342* (AMAZ, F, L). **San Martín**: Prov. Alto Amazonas, Yurimaguas-Tarapoto road, 15 km SW of Yurimaguas, 180 m, 10 Oct 1985, *Gentry et al. 52203* (MO, U).

#### Notes.

We have named this species after Sir Ghillean Prance, who enabled the first author (PM) to undertake his first steps in the Amazonian world and assisted and taught him in a very kind and generous way.

*Costussprucei* Maas and *C.prancei* sp. nov. closely resemble a third species, *C.chartaceus* Maas, which occurs in Amazonian Colombia, Ecuador, and Peru. They share many of the vegetative and floral features, but *C.chartaceus* Maas mostly has an unequally lobed ligule of 5–15 mm long, while that of the other two is truncate and 2–7 mm long. The essential difference can be found in the flower colour, which is pinkish-white in *C.chartaceus* (vs. pinkish-red in *C.sprucei* and yellow to orange in *C.prancei*).

The two Peruvian collections (*Gentry et al. 52203* and *Uliana et al. 1342*) look quite similar to this species and agree in most of the floral measurements, but the leaves are quite large for this species (i.e., 25–35 × 9–13 cm).

### ﻿ Costus pseudospiralis

Taxon classificationPlantaeZingiberalesCostaceae

Maas & H.Maas sp. nov.

73F68C6A-0B22-51BA-B9EF-7C273F37D7EA

urn:lsid:ipni.org:names:77316099-1

#### Diagnosis.

**Figure 18.**
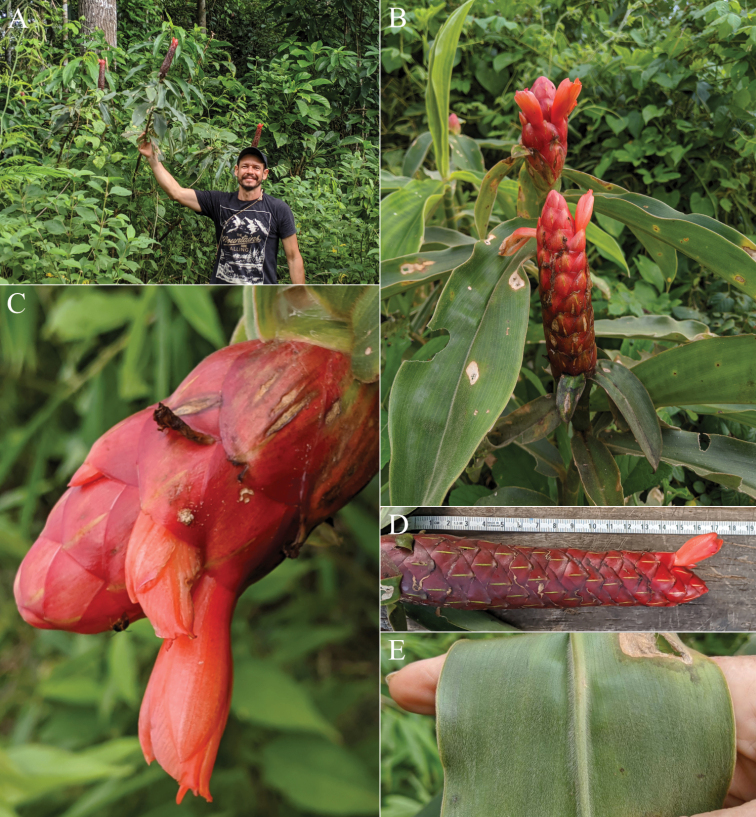
*Costuspseudospiralis* Maas & H.Maas **A** plant in habitat near Xapurí, Acre, Brazil with Martin Acosta **B** inflorescences emerging from the same rhizome **C** close-up of inflorescence with flowers showing their abaxial orientation **D** inflorescence with scale, emerging flower showing abaxial orientation **E** abaxial leaf surface showing indument. Photos **A–E** by Dave Skinner.

*Costuspseudospiralis* sp. nov. (Fig. 18) looks superficially quite similar to *C.spiralis* (Jacq.) Roscoe, but differs by the orientation of the flowers (floral opening facing abaxially v adaxially) and a cordate leaf base. It differs from *C.douglasdalyi* by the inflorescence terminating a leafy shoot instead of terminating a shorter leafless shoot (sometimes referred to as ‘basal’ in the literature), the presence of villose indumentum on most of its vegetative parts (vs. glabrous or sparsely puberulous), and a cordate (vs. acute to rounded) leaf base.

#### Type.

Bolivia, Beni: km 4 of road from Riberalta to Santa María, 150 m, 22 Jan 1999, *Maas*, *Maas-van de Kamer & Apaza 8751* (holotype U, 2 sheets: U1610003 & U1610004; isotypes K, MO, USZ).

#### Description.

***Herb*** 1.5–2 m tall. ***Leaves*** sheaths 10–15 mm diam; ligule obliquely truncate, 5–8 mm long; petiole 3–5 mm long; sheaths, ligule and petiole densely villose; lamina narrowly elliptic, 20–43 × 4–12 cm, adaxially densely to rather densely villose, abaxially densely villose, base cordate, apex acuminate (acumen 10–15 mm long). ***Inflorescence*** ovoid, 4–12 × 3–6 cm, terminating a leafy shoot; bracts, bracteole, calyx, ovary, and capsule glabrous. ***Flowers*** abaxially oriented; bracts red to dark red, coriaceous, broadly ovate, 2.5–3.5 × 2.5–3.5 cm, apex obtuse, callus 5–7 mm long, sometimes with a small, deltate appendage c. 5 × 5 mm; bracteole boat-shaped, 15–20 mm long; calyx red, 8–11 mm long, lobes shallowly ovate-triangular to very shallowly ovate-triangular deltate, 1–2 mm long; corolla dark salmon pink, 45–50 mm long, glabrous, lobes narrowly obovate-elliptic, 30–35 mm long; labellum pale salmon pink, apex and centre white, distal part of middle lobe yellowish, lateral lobes rolled inwards and forming a slightly curved tube 6–12 mm diam, oblong-obovate when spread out, 30–35 × 20–25 mm, apex irregularly 5-lobulate; stamen salmon pink, ca. 30 × 7 mm, not exceeding the labellum, apex white, rounded, anther 8–10 mm long. ***Capsule*** ellipsoid, 10–20 mm long.

#### Distribution.

Bolivia (Beni), Brazil (Rondônia) (Fig. 22G).

#### Habitat and ecology.

In secondary roadside vegetation, at elevations of 150–700 m. Flowering year-round.

#### Etymology.

This species is named “pseudospiralis”, referring to its resemblance to *C.spiralis*.

#### Paratypes.

**Bolivia. Beni**: 3 km E of Riberalta on road to Guayaramerín, then 2 km SE of side road, 230 m, 7 Jun 1982, *Solomon 7978* (L, LPB, MO). **Santa Cruz**: Prov. Velasco, Parque Nacional Noel Kempff Mercado, Los Fierros, 200 m, 8 Nov 1993, *Jardim & Quevedo 56* (MO, NY, USZ); Prov. Velasco, Parque Nacional Noel Kempff Mercado, Huanchaca II, 700 m, 4 Jul 1996, *Peña–Chocarro et al. 65* (U, USZ). **Brazil. Rondônia**: trail from Fortaleza to Rio Abuña, 20 km above mouth to São Sebastião mines, 15 Nov 1968, *Prance et al. 8459* (NY, U).

### ﻿ Costus rubineus

Taxon classificationPlantaeZingiberalesCostaceae

D.Skinner & Maas sp. nov.

191887D0-2981-5EFA-A87A-8804961FE2FE

urn:lsid:ipni.org:names:77316100-1

#### Diagnosis.

**Figure 19.**
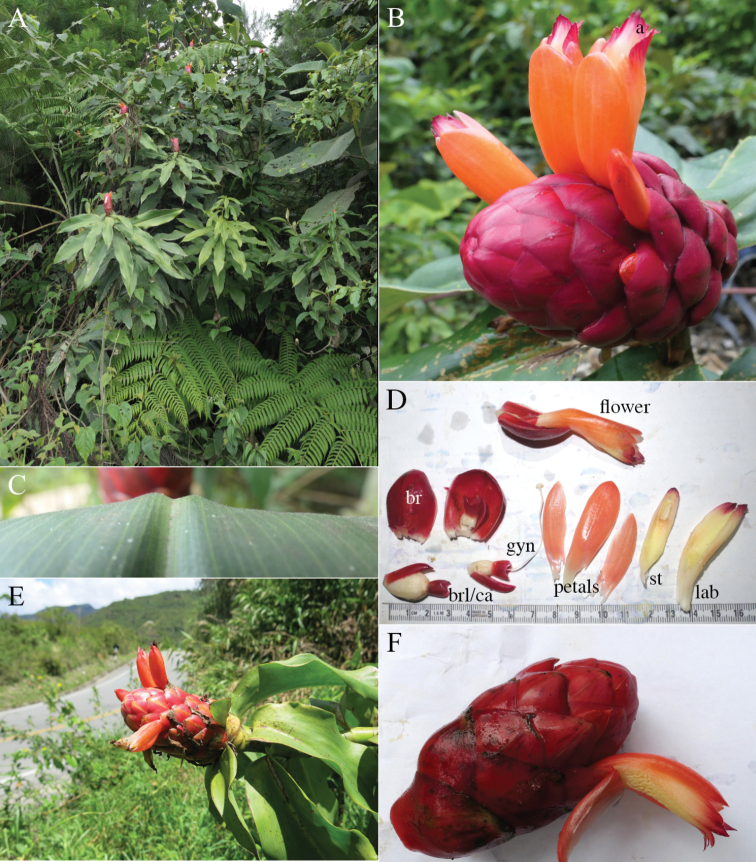
*Costusrubineus* D.Skinner & Maas **A** plant in habitat near Villa Rica, Pasco, Peru **B** inflorescence with flowers showing the labellum with characteristic lobulate apex (a) **C** adaxial leaf midrib showing absence of line of hairs **D** bract and flower detail from plant in habitat at Alto Churumazu, Pasco, Perú showing the full flower with subtending bract (flower), bract (br), bracteole subtending the capsule (ovary plus calyx) (brl/ca), stigma and style as attached to the young capsule (gyn), petals, single fertile stamen (st) and labellum (lab). Seeds from this plant were collected and cultivated as *D.Skinner R3421***E** plant in habitat at 1980 meters near Chinchao, Huánuco, Perú, the original collection site of “*C.ruber*” as indicated in Ruiz’ journal **F** inflorescence with lower bracts removed and single flower photographed in the wild near Chinchao, Perú. Photos **A–F** by Dave Skinner.

*Costusrubineus* sp. nov. (Fig. 19) looks somewhat similar to *C.scaber* Ruiz & Pav. but differs from that species by a larger calyx (9–15 vs. 3–7 mm), larger bracteole (20–28 vs. 9–12 mm), larger corolla (50–60 vs. 35–40 mm), and a labellum which is distinctly lobulate at the apex vs. entire in *C.scaber*; furthermore it is restricted to high elevations whereas *C.scaber* is mostly a lowland species; *C.rubineus* also lacks the row of hairs along the adaxial midvein of the lamina, which is so typical for *C.scaber*.

#### Type.

Peru, Pasco: Prov. Oxapampa, Gramazú, 1961 m, 17 Mar 2005, *Rojas G.*, *Vásquez Chávez & Francis 3574* (holotype L4196133; isotypes HUT, MO-2152325, USM).

#### Description.

***Herb*** 0.5–4 m tall. ***Leaves*** sheaths 15–30 mm diam; ligule truncate to slightly lobed, 2–7 mm long; petiole 3–8 mm long; sheaths, ligule and petiole densely sericeous to glabrous; lamina narrowly elliptic, 20–37 × 5–13 cm, adaxially glabrous, abaxially rather densely villose to almost glabrous, base obtuse to slightly cordate, apex acuminate (acumen 10–15 mm long) to acute. ***Inflorescence*** ovoid, 7–11 × 4–6 cm, terminating a leafy shoot; bracts, bracteole, and calyx rather densely puberulous to glabrous, ovary and capsule densely sericeous. ***Flowers*** abaxially oriented; bracts dark ruby red to red, coriaceous, broadly ovate, 3–4 × 2.5–4 cm, apex obtuse, callus 6–10 mm long; bracteole boat-shaped, 20–28 mm long; calyx red to pink, 9–15 mm long, lobes very shallowly triangular, 2–3 mm long; corolla pink to orange, 50–60 mm long, glabrous, lobes narrowly elliptic, 40–50 mm long; labellum yellow, distal part red, lateral lobes rolled inwards and forming a curved tube ca. 10 mm diam, oblong-obovate when spread out, 30–50 × 18–20 mm, 5-lobulate, lobules narrowly ovate-triangular, ca. 4 mm long; stamen yellow, 30–45 × 7–10 mm, not or slightly exceeding the labellum, apex red, irregularly dentate, anther 8–9 mm long ***Capsule*** obovoid, ca. 13 mm long.

#### Distribution.

Peru (Huánuco, Pasco) (Fig. 22H).

#### Habitat and ecology.

In primary or secondary, premontane or montane forests, at elevations of (300–)1400–2200 m. Flowering year-round.

#### Etymology.

*Costusrubineus* sp. nov. is named in reference to the Spanish explorer Hipolito Ruiz who visited in August 1780 the place called Chinchao in the Peruvian department of Huánuco. In his journal, he mentioned the collection of a plant he referred to as “*Costusruber*”, but his collection was lost in a shipwreck off the coast of Portugal, and this species’ name was never again mentioned in the journal. Since there is already a *C.ruber* C.Wright ex Griseb. (a synonym of *C.pulverulentus* C.Presl), we cannot use that name but are using the very apt name of *C.rubineus* to reflect the beautiful ruby-red colour of the bracts. The first author of this species visited the Chinchao region in 2016 and found at that locality specimen which he believed to be the same species as Ruiz mentioned in his journal (Skinner 2016).

#### Paratypes.

**Peru. Huánuco**: Cordillera Azul, ca. 42.7 km E of Tingo Maria on road to Pucallpa, 5650 ft, 21 Nov 1979, *Davidson & Jones 9395* (U). **Pasco**: Prov. Oxapampa, Distr. Huancabamba, Zona de Amortiguamiento del Parque Nacional Yanachaga-Chemillén, Sector alto tunqui, 1781 m, 10 Dec 2006, *Castillo et al. 642* (AMAZ, HUT, L, MO, USM); Prov. Oxapampa, Pichis Valley, new road between Santa Rose de Chivis and Puerto Bermúdez, 300–400 m, 30 Sept. 1982, *Foster 8950A* (MO); Prov. and Distr. Oxapampa, Carretera Oxapampa-Tsachopem, 1850 m, 15 Nov 2004, *Monteagudo Mendoza et al. 7658* (HUT, MO, U, USM); Prov. Oxapampa, Distr. Huancabamba, Carretera hacia Pozuzo-Zona de Amortiguamiento, Parque Nacional Yanachaga-Chemillén, 1390–1630 m, 22 Mar 2005, *Ortiz V. et al. 521* (L, MO, USM); Prov. Oxapampa, 5 km SE of Oxapampa, Oswald Müller property, 1850 m, 13–16 Dec 1982, *Smith 2946* (MO, U), idem, 24 Dec 1983, *Smith 5360* (HUT, MO, U, USM); Distr. Oxapampa, La Suiza, 10 Dec. 2002, 2200 m, *Vásquez Martínez & Rojas 27782* (F, HUT, MO, U, USM).

#### Notes.

*Costusrubineus* sp. nov. is a species mostly restricted to relatively high elevations (except for *Foster 8950A*, which was found at 300 m) in the departments of Pasco, San Martín, and Huánuco, Peru. It looks, at first glance, somewhat similar to *C.spiralis* (Jacq.) Roscoe, but it differs from that species by its abaxially oriented flowers and a very short ligule. The living plants look similar to *C.scaber* Ruiz & Pav., but that species differs by a much shorter calyx (3–7 mm vs. 9–15 mm long in *C.rubineus*) and the presence of a row of erect hairs on the adaxially of the lamina (lacking in *C.rubineus*). There are often several flowers at the same time at anthesis, whereas in *C.scaber*, there is usually just one flower at anthesis.

### ﻿ Costus whiskeycola

Taxon classificationPlantaeZingiberalesCostaceae

Maas & H.Maas sp. nov.

A8C88702-9884-54E3-B7F2-78BEECF5CEC4

urn:lsid:ipni.org:names:77316102-1

#### Diagnosis.

**Figure 20.**
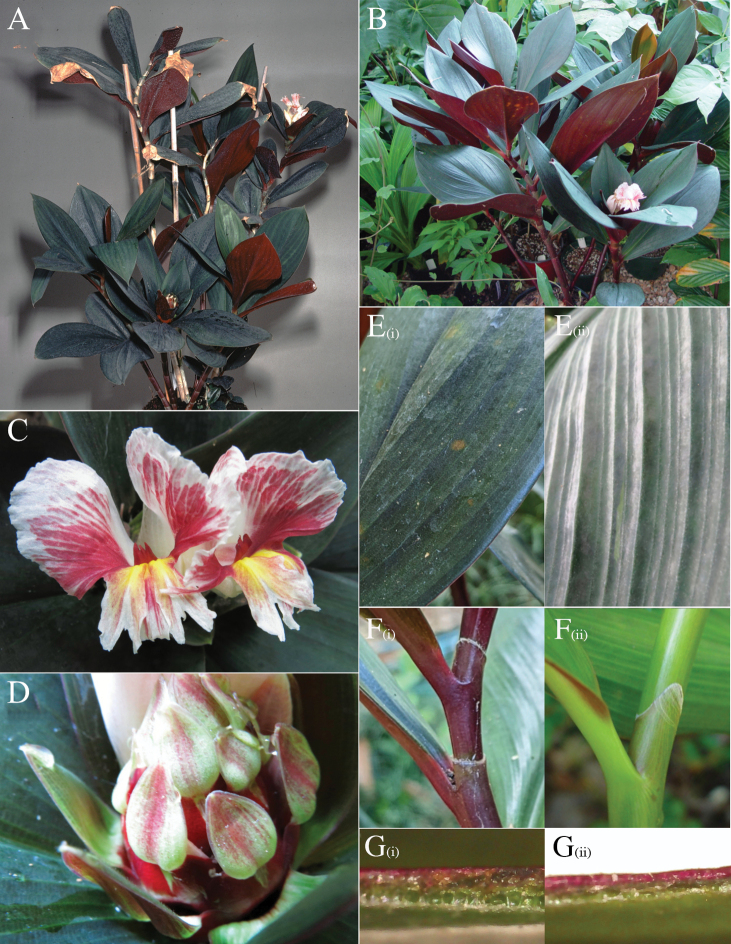
*Costuswhiskeycola* Maas & H.Maas **A** cultivated plant grown from seeds from the *Plowman & Davis 4396* accession now growing at the Utrecht Botanical Gardens, The Netherlands **B** cultivated plant obtained from the established nursery trade, accession *D.Skinner R2847***C** flowers showing spreading labellum with lateral markings and a prominent honey mark **D** bracts with green and red erect appendages **E** comparison of leaf plication between (i) *C.whiskeycola* and (ii) *C.erythrophyllus***F** comparison of ligule and leaf bases of (i) *C.whiskeycola* and (ii) *C.erythrophyllus***G** comparison of leaf sections showing thicker mesophil layer in (i) *C.whiskeycola* v. (ii) *C.erythrophyllus*. Leaf images in **C–D** taken from plants that were pressed and accessioned as *D.Skinner R3026* (C. *whiskeycola* sp. nov.) and *D.Skinner R2948* (*C.erythrophyllus*). Photo **A** by Lubbert Westra, photos **B–F** by Dave Skinner.

*Costuswhiskeycola* sp. nov. (Fig. 20) looks quite similar to *Costuserythrophyllus* Loes., sharing many features of inflorescence and flowers, but it is markedly different in their leaves which lack the distinct plication and which have a dark, olive green adaxial surface.

#### Type.

Colombia, Putumayo: Km 42 of Mocoa-Puerto Asis Road, Quebrada El Whiskey, Finca Santa Marta, Hilltop forest, c. 400 m (“1260 ft”), 5 Nov 1974, *Plowman & Davis 4396* (holotype COL; isotype PSO).

#### Description.

***Herb*** 0.5–0.7 m tall. ***Leaves*** sheaths 10–18 mm diam; ligule 10–20 mm long, obliquely truncate; petiole 5–15 mm long; sheaths, ligule and petiole glabrous, purple-red to green; lamina ovate-elliptic to narrowly ovate-elliptic, 22–29 × 10–13 cm, dark, olive green adaxially, red-purple to dark purple abaxially, with 5–6 dark green bands corresponding with slightly raised veins above, adaxial and abaxial surfaces both glabrous, base acute, apex acute to shortly acuminate (acumen 3–5 mm long). ***Inflorescence*** ovoid, ca. 7 × 4.5 cm, terminating a leafy shoot; bracts and appendages of bracts, bracteole, calyx, and ovary glabrous. ***Flowers*** abaxially oriented; bracts red, coriaceous, broadly ovate, 3–4 × 2–3.5 cm; appendages green, foliaceous, ascending, triangular-ovate, 2.5–5 × 2–3.5 cm, apex acute; bracteole boat-shaped, 24–30 mm long; calyx red, 12–20 mm long, lobes very shallowly triangular, 2–5 mm long; corolla white, 60–75 mm long, glabrous, lobes narrowly elliptic, 45–60 mm long; labellum white, distal edge horizontally spreading, broadly obovate, 60–70 × 50 mm, lateral lobes striped with red, middle lobe reflexed with yellow honey mark, irregularly lobulate, margin crenulate; stamen white, tinged with red, 35–40 × 13–14 mm, not exceeding the labellum, apex 3-dentate, anther 8–10 mm long. ***Capsule*** not seen.

**Figure 21.**
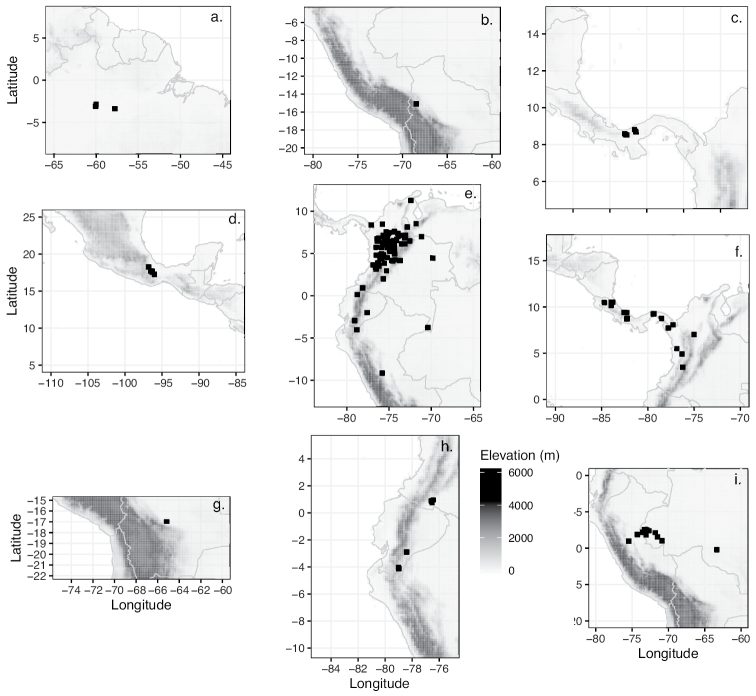
Distribution maps for new species of Costaceae: *Chamaecostus* – *Costusdouglasdalyi***A***Chamaecostusmanausensis* Maas & H.Maas **B***Costusalfredoi* Maas & H.Maas **C***Costusalleniopsis* Maas & D.Skinner **D***Costusalticolus* Maas & H.Maas **E***Costusantioquiensis* Maas & H.Maas **F***Costuscallosus* Maas & H.Maas **G***Costuscochabambae* Maas & H.Maas **H***Costusconvexus* Maas & D.Skinner **I***Costusdouglasdalyi* Maas & H.Maas.

#### Distribution.

**Figure 22.**
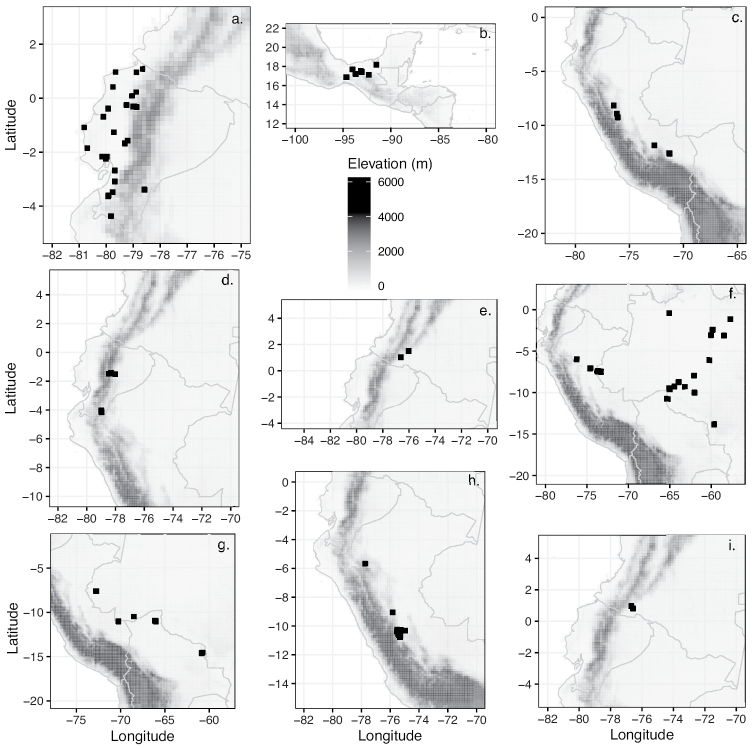
Distribution maps for new species of Costaceae. *Costusgibbosus – Costuswhiskeycola*m **A***Costusgibbosus* D.Skinner & Maas **B***Costusmollissimus* Maas & H.Maas **C***Costusobscurus* D.Skinner & Maas **D***Costusoreophilus* Maas & D.Skinner **E***Costuspitalito* C.D.Specht & H.Maas **F***Costusprancei* Maas & H.Maas **G***Costuspseudospiralis* Maas & H.Maas **H***Costusrubineus* D.Skinner & Maas **I***Costuswhiskeycola* Maas & H.Maas.

Colombia (Putumayo). Ecuador (Napo), Peru (Loreto) (Fig. 22I).

#### Habitat and ecology.

In tropical wet forests at an elevation of 250–400 m. Flowering period is uncertain.

#### Etymology.

This species has been named after its type locality El Whiskey (Putumayo, Colombia), with “cola” the Latin for “living in”.

#### Paratypes.

**Colombia. Putumayo**: El Chapo, 250 m, *Skinner R 3463*. **Cultivated Material** Greenhouse of “Sandwijck”, near Utrecht, the Netherlands, 3 Aug 1978, *Maas 74-618* (AAU, COL, K, NY, QCA, P, U161001, U161002, US), from the type collection *Plowman & Davis 4396*; cultivated in USBRG as 1994-680, *Skinner R 2257* (UC); cultivated in Jesse Durko Nursery, *Skinner R 2847*; cultivated by Bob Campos, *Skinner R 2948*; Smith College Botanical Garden, *Skinner R 3026* (UC).

#### Notes.

This new species has long been widely cultivated worldwide, originally grown from seeds collected by Tim Plowman when preparing *Plowman & Davis 4396* at the type locality. Until a late stage in our revision, we united this species with *C.erythrophyllus* Loes. Although the flowers and inflorescence look very much like those of that species, the leaves are quite different in their colour and the almost absence of distinct plication. They also have a thicker mesophil layer and are waxy in living plants. This species can also be distinguished from *C.erythrophyllus* by the length and shape of the ligules, which are truncate to obliquely truncate instead of being deeply lobed.

In April 2022, Dave Skinner visited the Santa Cruz private reserve of Project Amazonas near Iquitos, Loreto, Peru and found large populations of plants in primary forest that are identical vegetatively and have flowers that match those of *Costuswhiskeycola*. However, the bracts lack leafy appendages. It is yet to be determined whether or not these plants are of this same species.

## Supplementary Material

XML Treatment for Chamaecostus manausensis

XML Treatment for Costus alfredoi

XML Treatment for Costus alleniopsis

XML Treatment for Costus alticolus

XML Treatment for Costus antioquiensis

XML Treatment for Costus callosus

XML Treatment for Costus cochabambae

XML Treatment for Costus convexus

XML Treatment for Costus douglasdalyi

XML Treatment for Costus gibbosus

XML Treatment for Costus mollissimus

XML Treatment for Costus obscurus

XML Treatment for Costus oreophilus

XML Treatment for Costus pitalito

XML Treatment for Costus prancei

XML Treatment for Costus pseudospiralis

XML Treatment for Costus rubineus

XML Treatment for Costus whiskeycola

## ﻿Appendix 1

**Identification list**

Collections are identified by first collector and number only. The abbreviations behind the collector number refer to the following taxa:

Ch man = *Chamaecostusmanausensis* Maas & H.Maas

Co alf = *Costusalfredoi* Maas & H.Maas

Co all = *Costusalleniopsis* Maas & H.Maas

Co alt = *Costusalticolus* Maas & H.Maas

Co ant = *Costusantioquiensis* Maas & H.Maas

Co cal = *Costuscallosus* Maas & H.Maas

Co coc = *Costuscochabambae* Maas & H.Maas

Co con = *Costusconvexus* Maas & D.Skinner

Co dou = *Costusdouglasdalyi* Maas & H.Maas

Co gib = *Costusgibbosus* D.Skinner & Maas

Co mol = *Costusmollissimus* Maas & H.Maas

Co obs = *Costusobscurus* D.Skinner & Maas

Co ore = *Costusoreophilus* Maas & D.Skinner

Co pit = *Costuspitalito* C.D.Specht & H.Maas

Co pra = *Costusprancei* Maas & H.Maas

Co pse = *Costuspseudospiralis* Maas & H.Maas

Co rub = *Costusrubineus* D.Skinner & Maas

Co whi = *Costuswhiskeycola* Maas & H.Maas

Asplund 7833: Co ore; 15412: Co gib; 15895: Co gib; 16421: Co gib; 16494: Co gib; 16763: Co gib; 18160: Co gib; 20068: Co ore – Assunção 72: Co pra;

Balcázar 49: Co ant – Beck 7320: Co coc – Beltrán Cuartas 78: Co ant – Betancur B. 5970: Co ant; 7500: Co ant; 16507: Co ant; 16651: Co ant – Bilby 33: Co pra – Boeke 1009: Co ant – Bohlin 1226: Co gib – Boyle 2556: Co alt – Breedlove 26463: Co mol;

Calderón 2986: Co pra – Camp E 2395: Co ant – Campbell 8951: Co pra – Carreira 315: Co pra – Castro 81: Co pra – Castillo 642: Co rub – Cavalcante 540: Co pra; Cid 5437: Co pra – Cordero-P. 91: Co ant – Cornejo 425: Co gib; 5792: Co gib – Croat 62674: Co dou; 83948A: Co gib; 85008: Co dou; 92034: Co ant – Cuatrecasas 8396: Co ant; 13714: Co ant;

Daly 8606: Co dou – Davidson 8770: Co cal; 9395: Co rub – Dodson 913: Co ore; 1441: Co ore; 5347: Co gib; 13039: Co gib – Dressler 4190: Co cal – Duque-Jaramillo 2251: Co ant; 3912-A: Co ant;

Ehrich 5c: Co pra;

Fernández-Alonso 11440: Co pit – Fiaschi 3287: Co pra – Forero 5765: Co cal; 6161: Co ant; 6668: Co cal; 7413: Co ant – Foster 8950A: Co rub; 10937: Co obs – Frame 171: Co pra – Fuentes Claros 6216: Co alf – Funk 10490: Co cal;

Galeano 2036: Co ant – García-Barriga 18240: Co ant – Gentry 52203: Co pra; 72332: Co gib; 10165: Co gib; 27304: Co obs – Gilli 97: Co mol; 119: Co gib – Gómez Pignataro 21201: Co cal – Guevara-Ibarra 39: Co ant;

Hammel 15871: Co gib; 16513: Co cal; 17668: Co cal; 20494: Co cal; 20694: Co cal – Harling 14354: Co gib; 18358: Co gib – Haught 4292: Co ant – Hayden 1020: Co cal – Hernández G. 387: Co mol; 1435: Co mol – Hitchcock 20543: Co gib – Holm-Nielsen 27916: Co gib;

Idrobo 2027: Co ant – Ishiki 2400: Co mol;

Jardim 56: Co pse;

Kirkbride 1180: Co cal – Knapp 4519: Co cal; 5524: Co cal – Kress 86-1978: Co cal;

Labiak 6250: Co pra – La Frankie 85-38: Co cal – Lozano-Contreras 7397: Co ant – Lücking s.n.: Co cal;

Maas 596: Co ant; 1548: Co cal; 1652: Co all; 1783: Co ant; 2915: Co gib; 4795: Co gib; 8751: Co pse; 8999: Co dou; 10488: Co ant; 10631: Co ant; 10635: Co gib; 10688: Co cal; 10707; Co con; 10733: Co pit; P 12972: Co dou; P 13213: Co dou – MacDougal 4020: Co cal – Madison 4623: Co ant – Madsen 86796; Co con – Martínez Salas 3160: Co mol –Monteagudo Mendoza 7658: Co rub – Monteiro 601: Co dou;

Nagata 2811: Co gib – Nee 54842: Co pra – Nevling 1526: Co mol – Nuñez 306: Co gib – Nuñez V. 23785: Co obs;

Obando 284: Co ant – Øllgaard 90385; Co con – Ortiz V. 521: Co rub;

Peña-Chocarro 65: Co pse – Penland 179: Co ore – Pennell 8486: Co ant – Pennington 56 SD: Co gib – Plowman 1775: Co obs; 4454: Co gib; 4455: Co gib; 5054: Co obs; 5054 A: Co obs; 5791: Co obs; 7595: Co obs – Prance 3151: Co pra; 5218: Co pra; 6672: Co pra; 8227: Co pra; 8459: Co pse; 8866: Co pra; 11529: Ch man; 12230: Co dou – 12265: Co pra; 15300: Co pra – Proctor Cooper s.n.: Co pra – Pulido 107: Co ant;

Ramamoorthy 1790: Co mol – Ramos-Pérez 3052: Co ant – Rentería Arriaga 2123: Co ant – Rincón Guttiérrez 592: Co alt – Rivera Reyes 1610: Co alt – Rodrigues 2996: Co pra – Roe 928: Co mol – Rojas Gonzáles 3574: Co rub – Rojas-Zamora 136: Co ant – Rubio 2007: Co gib;

Schunke V. 10195: Co ant; 15567: Co dou; 16185: Co dou – Silveira 559: Co pra; 596: Co pra – Silverstone-Sopkin 3085: Co ant; 5811: Co ant; 8963: Co ant – Skinner 3322: Co obs; R 3131: Co rub; R 3332: Co ore; R 3333: Co ore; R 3833: Co obs – Smith 2946: Co rub; 5360: Co rub – Solheim 1367: Co alt – Solomon 7978: Co pse – Sparre 13862: Co gib –

Steward 476: Co pra;

Uliana 1342: Co pra – Ulloa U. 111: Co gib;

Vareschi 1674: Co ant – Vargas-Figueroa 855: Co ant – Vásquez Martínez 27782: Co rub – Vélez J. 7073: Co ant – Vieira 369: Co dou – Villanueva 1844: Co ant – Von Martius s.n.: Co pra;

Wessels Boer 2440: Co ant.
